# Molecular exploration of paediatric intracranial germinomas from multi-ethnic Singapore

**DOI:** 10.1186/s12883-020-01981-0

**Published:** 2020-11-14

**Authors:** Sharon Yin Yee Low, He Cheng, Ruiyang Zou, Lee Ping Ng, Chik Hong Kuick, Nurfarhanah Bte Syed Sulaiman, Kenneth Tou En Chang, David Chyi Yeu Low, Lihan Zhou, Wan Tew Seow

**Affiliations:** 1 Neurosurgical Service, KK Women’s and Children’s Hospital, 100 Bukit Timah Road, Singapore, 229899 Singapore; 2 Department of Neurosurgery, National Neuroscience Institute, Singapore, Singapore; 3 SingHealth Duke-NUS Neuroscience Academic Clinical Program, 11 Jalan Tan Tock Seng, Singapore, 30843 Singapore; 4 VIVA-KKH Paediatric Brain and Solid Tumours Laboratory, Singapore, Singapore; 5 Bioprocessing Technology Institute, A*STAR, Singapore, Singapore; 6 MiRXES Pte Ltd, 10 Biopolis Road, Chromos, Singapore, 138670 Singapore; 7 Department of Pathology and Laboratory Medicine, KK Women’s and Children’s Hospital, 100 Bukit Timah Road, Singapore, 229899 Singapore

**Keywords:** Intracranial germinomas, KIT, miRNA

## Abstract

**Background:**

Germinomas (IG) account for up to 50% of all intracranial germ cell tumours. These tumours are reputed to be more prevalent in Oriental populations in comparison to Western cohorts. Biological characteristics of IG in other ethnic groups are unknown. Singapore is a multi-ethnic country with diverse cultures. Owing to inter-racial heterogeneity, the authors hypothesize there are molecular differences between paediatric IG patients in our local population. The aims of this study are exploratory: firstly, to identify molecular characteristics in this tumour type and circulating CSF unique to different racial cohorts; and next, to corroborate our findings with published literature.

**Methods:**

This is a single-institution, retrospective study of prospectively collected data. Inclusion criteria encompass all paediatric patients with histologically confirmed IG. Excess CSF and brain tumour tissues are collected for molecular analysis. Tumour tissues are subjected to a next generation sequencing (NGS) targeted panel for *KIT and PDGRA*. All CSF samples are profiled via a high-throughput miRNA multiplexed workflow. Results are then corroborated with existing literature and public databases.

**Results:**

In our cohort of 14 patients, there are *KIT* exon variants in the tumour tissues and CSF miRNAs corroborative with published studies. Separately, there are also *KIT* exon variants and miRNAs not previously highlighted in IG. A subgroup analysis demonstrates differential CSF miRNAs between Chinese and Malay IG patients.

**Conclusion:**

This is the first in-depth molecular study of a mixed ethnic population of paediatric IGs from a Southeast Asian cohort. Validation studies are required to assess the relevance of novel findings in our study.

**Supplementary Information:**

The online version contains supplementary material available at 10.1186/s12883-020-01981-0.

## Background

Intracranial germ cell tumours are a diverse group of lesions that are thought to arise from nests of primordial germ cells that instead of migrating towards the developing gonads, aberrate to the central nervous system (CNS) [1]. Within the germ cell tumour subtypes, intracranial germinomas (IG) account for up to 41 to 50% of all such tumours [2]. For uncertain reasons, these tumours are more prevalent in Oriental populations [3, 4], accounting for up to 11% of all paediatric brain tumours [5]. Conversely, they make up to 0.4 to 3.4% for the same disease in the Western cohort [6, 7]. Owing to this racial predilection, several IG-related studies are based on Far Eastern populations [6, 8, 9]. The proto-oncogene c-KIT (*KIT*), which is highly expressed in all IG, encodes a transmembrane tyrosine kinase receptor for stem cell factor that is related to the platelet-derived growth factor receptor A (*PDGFRA*) [10]. Advancements in genomic technology has offered significant insights into IG biology. These include the identification of various *KIT* mutations and specific miRNAs that may harbour functions in IG tumorigenesis [11–13]. Despite such noteworthy efforts, knowledge gaps remain in the management of IG patients. Firstly, given the racially homogeneous results from previous studies [12, 14, 15], molecular characteristics of IGs from non-Oriental ethnic populations are still unknown. Next, in comparison to other subtypes of germ cell tumours, diagnostic blood and cerebrospinal fluid (CSF) biomarkers in IGs remain elusive *hic et nunc*.

Singapore is a multi-ethnic island city-state with diverse cultures. She serves as a microcosm of East, South and Southeast Asia ethnic groups [16]. Her people consist of Chinese (74.1%), Malay (13.4%), Indians (9.2%) and other races [17]. The KK Women’s and Children’s Hospital is Singapore’s largest specialist paediatric hospital. In clinical practice, IG is the most common germ cell tumour encountered. Owing to inter-racial heterogeneity, the authors hypothesize there are molecular differences between paediatric IG patients in our local population. Building on this, the aims of this study are exploratory: firstly, to identify potential clinical and molecular characteristics in the tumour and CSF unique to different racial cohorts; and next, to corroborate our findings with published literature.

## Methods

### Study design and patient demographics

**Fig. 1:**
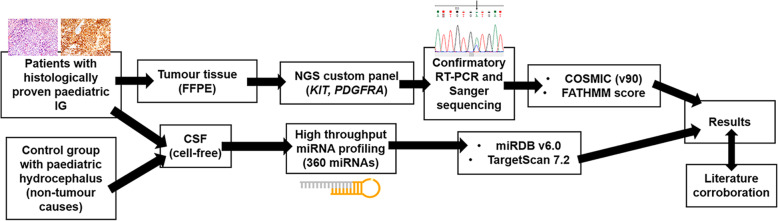
Summary workflow of the study conducted. (FFPE = formalin-fixed paraffin-embedded; NGS = next generation sequencing; RT-PCR = real time polymerase chain reaction; COSMIC = Catalogue Of Somatic Mutations In Cancer; FATHMM = Functional Analysis through Hidden Markov Models)

This is an ethics-approved, single-institution, retrospective study of prospectively collected data (Singhealth CIRB 2011/314/A). All patients and their legal guardians provided informed consent for the research use of their medical data and biomaterials. Inclusion criteria encompass all patients less than 18 years old who have histologically confirmed IGs from surgical specimens, under the care of the Neurosurgical Service, KKH. A separate group of patients with hydrocephalus secondary to non-neoplastic conditions who require CSF diversion surgery are recruited under the control arm. All CSF samples are taken from consented subjects at the time of surgery. Upon collection, each CSF sample is centrifuged, and its supernatant extracted to maintain a cell-free state. Excess brain tumour tissue taken during the same surgery is collected. For the patients in the control group, they only had CSF samples collected, as their surgical interventions did not require any brain tissue removal. Exclusion criteria included patients with incomplete medical data, insufficient or poor-quality CSF and, or inadequate tumour tissue. Figure 1 provides an overview of the study’s workflow.

### Next-generation sequencing

Ampliseq targeted gene sequencing panel comprising of *KIT* (NM_000222) and *PDGFRA* (NM_006206) is designed using Ion Ampliseq designer tool (version 6.0) (https://www.ampliseq.com) with the coverage of 100 and 97.69% respectively. Ten ng of FFPE DNA is used for automated library preparation using the Hi-QTM view chef DL kit (Life Technologies, USA) and the Ion chef (Life Technologies, USA). The library is enriched using the HI-QTM view Chef kit (Life Technologies, USA) in the Ion Chef. Eight libraries are multiplexed in an Ion 314 chip and sequenced in the ion-torrent sequencer (Life Technologies, USA). Base calling and mapping (hg19) are performed in the Torrent Suite (version 5.0), and Variant Calling plugin was applied to generate variant call format files. Variant annotation is done online wANNOVAR (http://www.wannovar.wglab.org). Only exonic variants with minimum 10% of variant allele frequency are selected for further validation. Confirmatory real-time polymerase chain reaction (RT-PCR) and subsequent Sanger Sequencing are performed to confirm NGS findings.

### High throughput miRNA profiling

This method has been previously described [18, 19]. Briefly, Qiagen miRNeasy Serum/ Plasma kit (Qiagen, Netherlands) is used to isolate miRNA from CSF. Isolation spike-in (3 synthetic small RNAs) is added in Proteinase K buffer and QIAzol during CSF isolation. Next, isolated total RNAs are reverse-transcribed using the IDEAL miRNA reverse-transcription (RT) kit (MiRXES, Singapore) and modified stem-loop RT primer pools. The reaction mixtures are incubated in a Veriti™ Thermal Cycler (Applied Biosystems, USA) at 42 °C for 30 min, followed by 90 °C for 5 min. For all miRNA profiling experiments conducted in this study, a 6-log serial dilution of synthetic templates (10^7 to 10^2 copies) for each miRNA and a non-template control are concurrently reversed-transcribed along with the isolated total RNA. Following that, all cDNAs are pre-amplified through a 14-cycle PCR reaction using the Augmentation Master Mix (MiRXES, Singapore) and Augmentation Primer Pools (MiRXES, Singapore) in the Veriti™ Thermal Cycler. A total of 360 candidate miRNAs (miRBase-annotated) were measured in each amplified cDNA sample using miRNA-specific quantitative PCR assays (MiRXES, Singapore). Each amplified cDNA sample is diluted 100 times in poly-A water. Five μl of diluted cDNA was then transferred into each well on a 384-well PCR plate. Polymerase chain reaction amplification reactions are performed in a total reaction volume of 15 μl that contains 5 μl of diluted cDNA, 7.5 μl of IDEAL SYBR Green qPCR Master Mix (2x, MiRXES, Singapore), 1.5 μl of miRNA specific qPCR primer pair (10x, MiRXES, Singapore)) and 1 μl of nuclease-free water. Quantitative PCR (qPCR) amplification and detection on QS5 qPCR system (Applied Biosystems, USA) is performed with the following cycling conditions: 95 °C for 10 min, followed by 40 cycles of 95 °C for 10 s and 60 °C for 30 s. Raw Cycle Threshold (Ct) values are read from the ViiA 7 RUO software with automatic baseline setting and a threshold of 0.5. RT-qPCR efficiency. Next, potential cDNA amplification bias are assessed by analysing the Ct values of serially diluted synthetic miRNA templates, which are concurrently reverse-transcribed, amplified and detected with the isolated CSF miRNAs. Absolute quantification of miRNA expression copy numbers is achieved through interpolation of synthetic miRNA standard curves. Copy numbers interpolated from standard curves are normalized by spike-ins before other downstream processing. Obtained copy numbers are then compared against non-template controls. When copy numbers are lower than non-template controls, the miRNA in the sample is considered a non-detect. Biological normalization is performed. Copy Numbers are log_2_ transformed and the mean of all miRNAs in all samples is levelled. Normalized miRNA expression values are used to compare the expression levels of individual miRNAs between IG patients and controls. Unsupervised hierarchical clustering was carried out based on Euclidean distance.

### Statistical analysis of miRNA results

The following methodology has been previously described [20]. To summarise, foldchange in absolute miRNA expression (copy number is standardized using a *z*-score {standard score} is calculated using the formula: *z*-score = log_2_ (FC/SD); where FC is the foldchange of miRNA expression between IG and control patients, and SD is standard deviation of expression levels for each miRNA. Differences between sample means (i.e. changes in miRNA expression) are considered statistically significant when the *p-*value is less than 0.05 via Student’s t test. All *p*-values are 2-sided and corrected for multiple hypothesis testing using the FDR adjustment [21, 22]. All statistical analyses are performed using MATLAB Toolbox (MathWorks, USA).

### Predictive analysis of miRNA-mRNA mapping

To facilitate the process of selecting functional targets, online interactive resources are used for individual miRNA target prediction and functional annotations. These included miRDB version 6.0 (http://www.mirdb.org), and TargetScan 7.2 (http://targetscan.org). The top 50 predicted mRNA targets from each database are cross-referenced against each other, and subsequently, matched to common genes reported in germ cell tumours (*KIT, PLAP, OCT 3/4, KLF4* and *NANOG*) [23].

## Results

### Overview of patient demographics and clinical features

A total of 19 patients were recruited for this study from 01 January 2009 to 31 December 2017. Fourteen (10 males and 4 females) were under the investigative arm and 5 (3 males and 2 females) were in the control group. For the 14 patients, all of them underwent surgical biopsies and had histologically proven IGs. Brain tumour specimens from these patients had their diagnosis of IG reviewed in accordance to the latest WHO classification [6]. Every patient had serum and CSF levels negative or inconclusive for αFP and βhCG. Their ages ranged from 7 to 15 years old (median age 12.4 years old). Nine were Chinese and 5 were of Malay ethnicity. Interestingly, we did not encounter Indians or other racial groups with IGs at our institution. In our study cohort, 6 had their tumours located in the pineal region, 3 suprasellar, 2 basal ganglia, 2 intraventricular and 1 in the cerebral peduncle. Evidence of tumour seeding was demonstrated in 6 patients. The latter was confirmed via CSF cytological positivity for malignant cells and, or radiological presence of metastasis distant from the primary tumour. Separately, there were 2 patients with tumour recurrence in our study. Both had initial suprasellar tumours that were treated successfully. One presented with tumour recurrence in the same site 8 years later and the other presented with neck pain secondary to a intramedullary IG in her cervical spine 10 years later. For both patients, surgical biopsy was performed. Histology reported features of IG, consistent with their earlier suprasellar tumours. These findings are summarised in Table 1.

**Table 1:** Summary of study cohort’s patient clinical and demographical details

Variable		Number of patients
**Gender**	**Male**	10
**(IG patients)**	**Female**	4
**Gender**	**Male**	3
**(Control group)**	**Female**	2
**Ethnicity**	**Chinese**	9
	**Malay**	5
	**Others**	0
**Age**	**IG patients**	14 (range 7 to 15 years old; median 12.4 years old)
	**Control group**	5 (range 1 month to 7 years old; median 1.62 years old)
**Location of tumour**	**Pineal**	6
	**Suprasellar**	3
	**Basal ganglia**	2
	**Intraventricular**	2
	**Cerebral peduncle**	1
**Tumour seeding**	**Yes**	6
**(at initial diagnosis)**	**No**	8
**Tumour recurrence**	**Yes**	2
**(at ≥ 5 years)**	**No**	12
**Serum αFP (≤ 8 μg/L)**	**< 2 μg/L**	11
	**≥ 2** **μg/L**	3 (range 3 to 5 μg/L; median 3.67 μg/L)
**Serum βhcg (< 5 IU/L)**	**< 1.2 IU/L**	9
	**≥ 1.2 IU/L**	4 (range 1.4 to 14 IU/L; median 6.6 IU/L)
**CSF αFP (< 2 μg/L)**	**< 2 μg/L**	14
**CSF βhcg (< 1.2 IU/L)**	**< 1.2 IU/L**	6
	**≥ 1.2 IU/ L**	8 (range 1.6 to 20 IU/L; median 9.32 IU/L)

### Next generating sequencing identifies *KIT* mutations in a subset of patients in our study cohort

Based on NGS testing, 6 out of 14 patients reported *KIT* variants that were confirmed to be pathogenic on COSMIC (https://cancer.sanger.ac.uk/cosmic). Of interest, 5 of them (3 Malay and 2 Chinese) harboured *KIT* exon 10 variant (c.1621A > C). Two patients had more than coding variant in different *KIT* exons in their tumours. This included a Chinese patient who had *KIT* exon 2 (c.251C > T) and exon 17 (c.2447A > T) variants; and another Malay male who was found to have variants in *KIT* exon 10 (c.1621A > C), exon 11 (c.1658A > G) and exon 13 (c.1965 T > A). Conversely, all of our patients did not show any meaningful *PDGFRA* exonic variants from the NGS interrogation (Table 2).

**Table 2:** Summary of *KIT* exonic variants from NGS results after cross-referencing from COSMIC. Of note, one patient had 2 *KIT* variants, and another had 3 *KIT* variants

*KIT*	AA mutation	CDS mutation	FATHMM score	COSM identifier	Reported in human cancers (Yes or No)	Number of patients with variant
Exon 2	p.T84M	c.251C > T	0.01 (neutral)	COSM3380948	Yes; mixed germ cell tumour, pancreatic cancer	1
Exon 10	p.M541L	c.1621A > C	0.74	COSM28026	Yes; primary CNS lymphoma, breast cancer	5
Exon 11	p.Y553C	c.1658A > G	0.96	COSM4413463	Yes; mixed germ cell tumour	1
Exon 13	p.N655K	c.1965 T > A	0.82	COSM4413464	Yes; germ cell tumour, AML	1
Exon 17	p.D816V	c.2447A > T	0.99	COSM1314	Yes; germ cell tumour, malignant melanoma	1

### Cerebrospinal fluid in intracranial germinoma patients express miRNAs that are both novel and corroborative with published literature

**Fig. 2:**
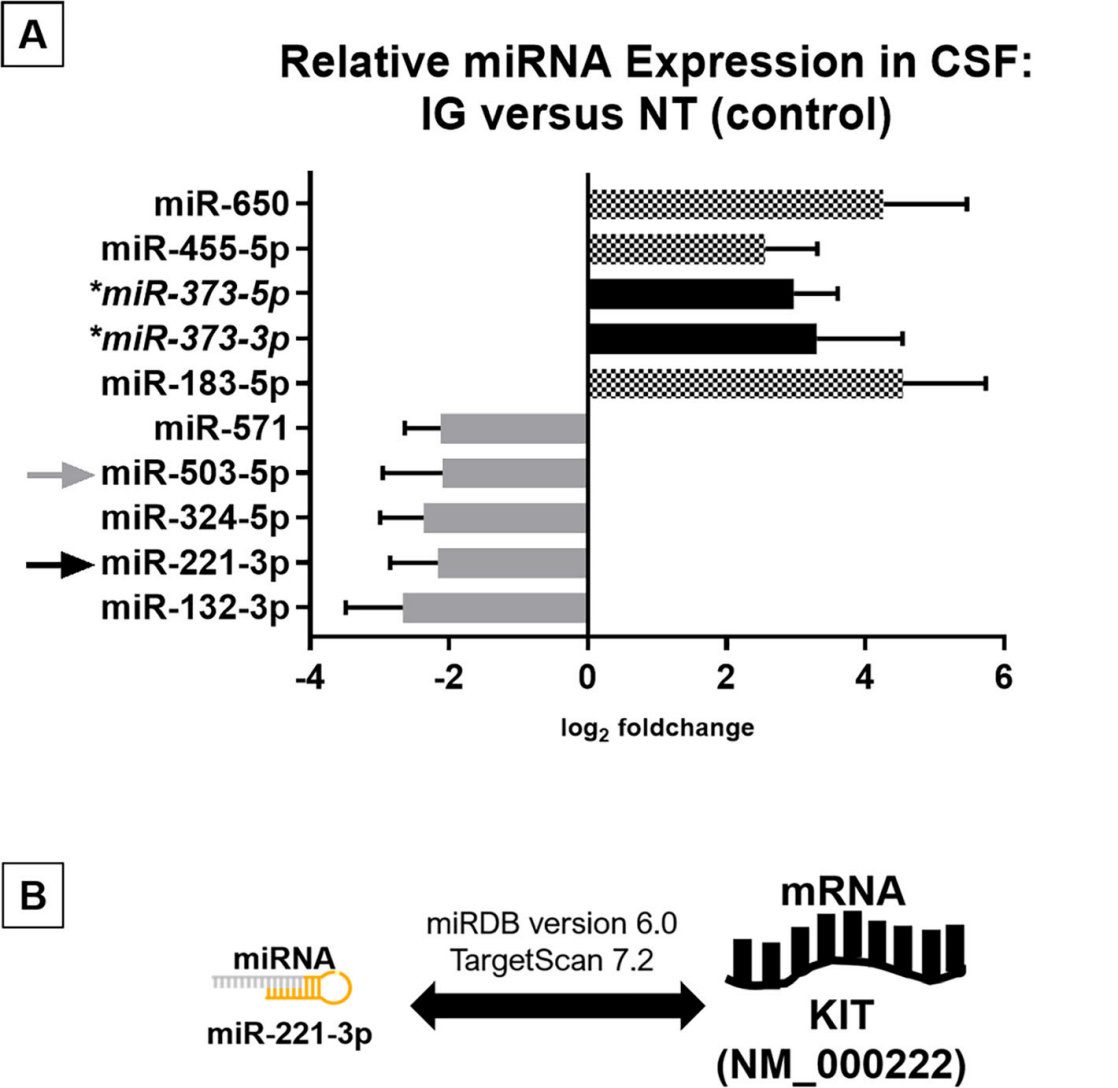
**a** Graph illustration of differentially expressed miRNAs in the CSF between IG patients and non-tumour (NT) controls. Of note, miR-373 has been previously reported to have higher expression in patients with germ cell tumours [13]. In addition, mir-503-5p has been shown to have a lower expression in the tissue of germinomas compared to non-germinatous tumours [23]. **b** Using online databases (miRDB version 6.0 and TargetScan 7.2), in silico prediction reports that mir-221-3p is associated with *KIT*

For the CSF miRNA profiling, our results showed statistically significantly higher expression of miR-373-3p, miR-373-5p, miR-455-5p, miR-650 and miR-183-5p; and lower expression of miR-571, miR-503-5p, miR-324-5p, miR-221-3p and miR-132-3p for the IG cohort, in comparison to the control group. The remaining miRNAs in the multiplex panel had negative or equivocal findings for the same analysis. (Fig. 2a).

### MiR-221-3p is downregulated in intracranial germinomas and is a predicted target of *KIT*

Owing to the inverse relationship in the miRNA-mRNA connection, downregulation of a miRNA implies an increase in expression of its related mRNA, either via direct or indirect regulation. Our results show that miR-221-3p has a significantly lower expression for IG patients in comparison to the control group. Based on in silico prediction, mir-221-3p is found to be consistently associated with *KIT* using miRDB version 6.0 and TargetScan 7.2. (Supplementary Data A and B, respectively). At this point in time, a validated link between miR-221-3p and *KIT* is not yet established in germ cell tumours. The remaining miRNAs did not show any correlation with the established genes of interest in IG. (Fig. 2b).

### Subgroup analysis shows some differences in CSF miRNA expression between Chinese and Malay patients

**Fig. 3:**
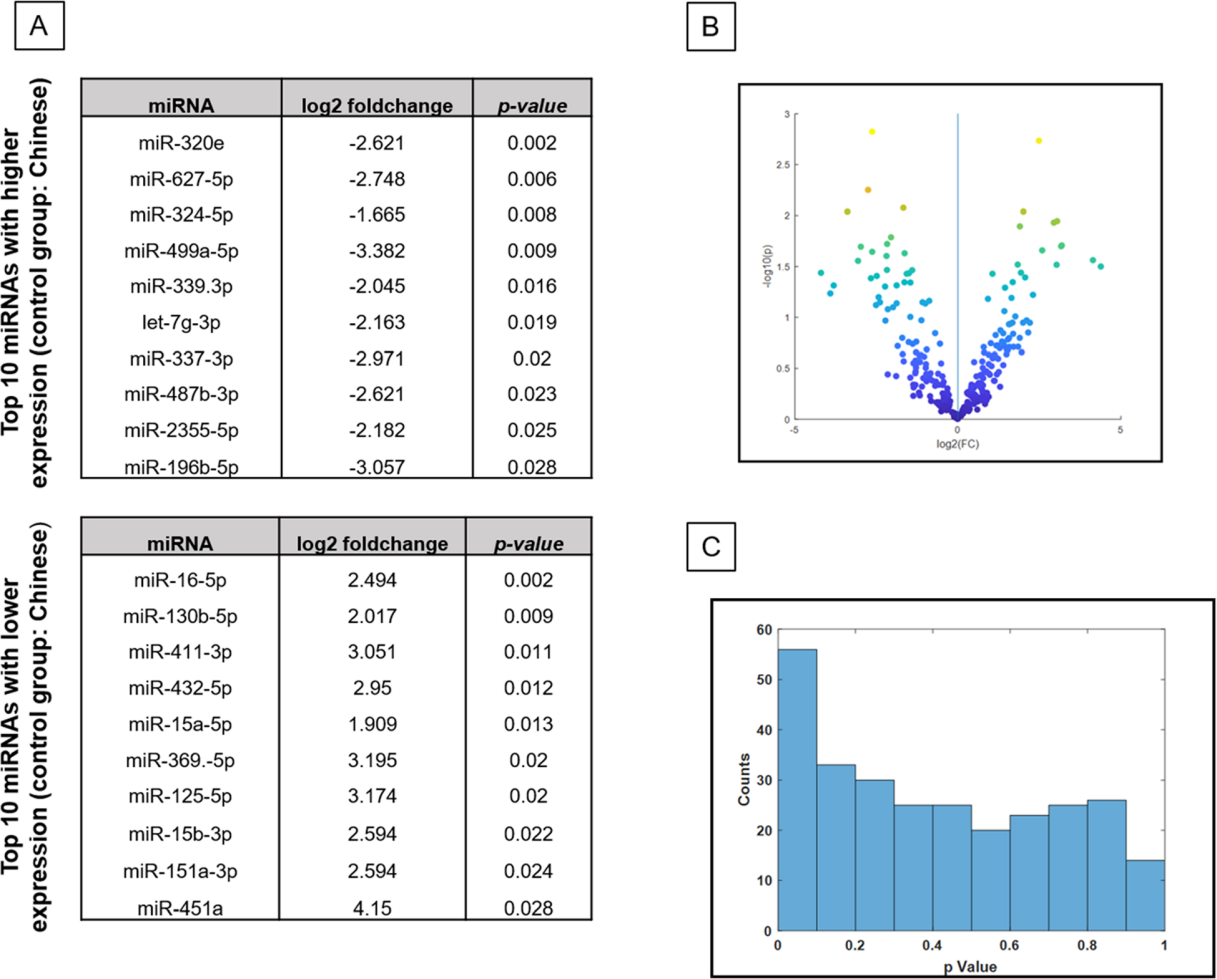
**a** Top 10 highest and lowest differentially expressed miRNAs between Malay and Chinese patients. **b** Volcano plot of expression differences between Malay and Chinese patients for all the profiled miRNAs. **c** Left-skewed histogram illustrating degree of overall difference between Malay and Chinese patients, based on statistical analysis of miRNA expression

A separate in-depth analysis of the CSF miRNA profiles between the Chinese and Malay IG patients was performed. Results showed there were a total of 39 miRNAs differentially expressed between both ethnic groups that were statistically significant. (Fig. 3).

## Discussion

### Intracranial germinomas: knowledge gaps

At present, there are 2 clinical gaps in the management for IGs: firstly, it is a tumour that does not express highly elevated βhCG and, or αFP levels in the patient’s serum or CSF. Surgical biopsy is usually recommended for diagnosis, and to exclude it from other lesions, such as teratoma. For most patients, the deep, midline structures, such as the pineal or suprasellar regions are most commonly affected [1]. Although histological diagnosis is advocated prior to adjuvant treatment, many global centres practice a trial of radiotherapy to the cranial lesion if the radiological findings, patient demographics and, or clinical presentation is convincing [24, 25]. This is because deeply seated tumours, especially those in the pineal region are close to critical vascular and brain structures. Historically, operative morbidity for lesions in such locations is notoriously high [26–29]. Taken together, a *specific* circulating biomarker for IGs will be diagnostically relevant for affected patients. Next, there is a paucity of established pre-clinical models commercially available for IG research. At the time of this writing, there is only 1 published study involving the successful establishment of patient-derived IG cell lines in the laboratory [30]. As part of the effort to overcome such knowledge gaps, ongoing work in our laboratory is underway to develop such translationally relevant models, especially to elucidate the relationship demonstrated by our in silico findings.

Overall, biological factors responsible for the different ethnic incidences of IG are poorly understood [31]. Current evidence suggests that IGs, together with other types of intracranial germ cell tumours, tend to be more common in people with Asian/ Oriental descent [3, 32, 33]. In Singapore, various epidemiological studies have reported inter-racial differences between ethnic groups in the prevalence, tumour biology, treatment patterns and patient survival for several diseases, including cancers [34–38]. Building on these observations, the authors postulate that molecular variations possibly exist between ethnic populations within our study cohort. Although our preliminary findings suggest there are differentially expressed miRNAs between our Chinese and Malay patients, we reiterate that confirmatory investigations in a larger Southeast Asian population are necessary to confirm their clinical significance. Research collaborations with our neighbouring countries are included in the future work of this study.

### Identification of KIT mutations in intracranial germinomas

The *KIT* gene encodes the stem cell growth factor receptor, a type III transmembrane receptor tyrosine kinase that has been involved in the pathogenesis of various malignancies, including gastro-intestinal stromal tumours, acute myelogenous leukaemia and testicular seminomas [39–41]. *KIT* signalling is upstream of *RAS-MAPK* signalling and the *PI3K* pathway. Activating mutations in *KIT* and other genes in *MAPK* and *PI3K* pathways are the most common genomic variations found in IG [42, 43]. Furthermore, a recent targeted sequencing study confirms that activating mutations in *KIT* and *RAS* are frequent and mutually exclusive in pure germinoma, suggesting that changes in the *KIT* signalling pathway plays an important role in the development of IG [44]. Aside from gain-of-function mutations of *KIT* in approximately 25% of IG, little is known about other oncogenic factors that partake in its pathogenesis [45, 46]. From a clinical perspective, this is significant as up to 10% of IG remain refractory to standard therapy [8]. In congruency with publications on IGs, our cohort too, has IG patients with *KIT* mutations in exon 11 and 17 [46]. However, *KIT* exon 10 (c.1621A > C) is the most common variant in our study group. Based on COSMIC referencing, this variant has been previously reported in primary CNS lymphoma [47] and brain metastases from breast carcinoma [48]. To our knowledge, this is the first report of *KIT* mutations involving paediatric IG of Malay ethnicity.

Recent advances in genomic technology have enabled detection of circulating tumour DNA (ctDNA) in the CSF of patients with CNS tumours [49, 50]. Circulating tumour DNA refers to the DNA that directly comes from tumour cells and stably circulates in body fluids [51]. Previous reports have demonstrated that ctDNA with a series of gene mutations can be successfully extracted from CSF of patients with brain tumours [49]. Under such circumstances, it is attractive to extrapolate the same approach to our paediatric IG patients. A word of caution though, there are noteworthy challenges for this process that need to be addressed. To begin with, absolute quantities of ctDNA in the CSF are reputed to be low. This implies that a significantly higher volume of CSF is required per patient which may be difficult to obtain from young children. Furthermore, highly sensitive methods such as WGS and NGS require additional technical and bioinformatic approaches to facilitate enhanced the ability to detect tumour mutations in CSF cfDNA. Hence, they can be costly and time-consuming [52]. Although there is an alternative option of using droplet digital PCR, users require prior knowledge of the specific mutations they want to test for, and the technology may be only limited up to 4 targets per assay [53]. Nonetheless, the development of a targeted panel for CSF ctDNA to complement our miRNA findings is certainly a consideration in time to come.

### Role of miRNAs in intracranial germinomas: towards disease diagnosis and better understanding?

MicroRNAs (miRNAs) are a class of small, noncoding RNA molecules typically 22 nucleotides in length [54]. They modulate protein expression through base pairing with a complementary sequence in the 3′-untranslated region of messenger RNA (mRNA) and are involved in the regulation of gene expression at a post-transcriptional level [55]. Established roles of miRNAs include the regulation of important processes including tumorigenesis [56–58]. In contrast to mRNA and proteins, they are inherently stable and remain conserved under most circumstances [59]. Circulating miRNAs in body fluids have been extensively explored as novel biomarkers for various diseases [60]. For instance, Murray et al show that certain miRNAs can be reliably detected in CSF as biomarkers for germ cell tumours [13]. Coincidently, his series included 2 IG patients that showed high expression of miR-373-3p in their CSF samples, which was also demonstrated in our study cohort. Next, Wang et al’s publication reports differentially expressed miRNAs between IGs and non-germinomatous germ cell tumours. In his study, miR-503 is one of the miRNAs with low expression in IG [23]. The downregulation of miR-503-5p is also observed in our study. Nonetheless, the remaining miRNAs, including miR-221-3p that was predicted to be a target of *KIT*, require deeper functional assessment and clinical validation.

The inherent properties of miRNAs deem them highly desirable for use as disease markers; hence, initiating the interest in the expression of profiles of miRNAs. MicroRNA profiling represents an important first-step in deducting individual RNA-based regulatory function in cells, tissues and circulation [61]. Nevertheless, there is no standardized clinical-grade platform for detection of circulating miRNAs. At present, real-time quantitative PCR remains as the most sensitive method for the quantification of the RNA species [62, 63]. Our project utilizes an innovative hemi-nested real-time quantitative PCR multiplex assay which is simple to design, shows excellent performance and provides design flexibility of any miRNA [64]. As our study is exploratory at this point, the use of a such a corroborative platform offers advantages of high throughput, sensitive and accurate examination of small CSF sample volumes. Such endeavours are particularly important in a limited patient cohort, like ours.

### Study critiques and future directions

The authors acknowledge that there are limitations that should be highlighted. First and foremost, this is a retrospective study with a small patient number. This is inevitable as data completeness, sufficient biomaterial per patient and adherence to a strict criterion for the purposes of this multimodality study are required. Owing to the infrequency of surgical biopsy and the small size of specimens (even if biopsies are performed), studies involving molecular and cytogenetic characteristics of IG are comparatively less common [31]. This challenge is also experienced in our study, whereby most of our cohort’s biopsies are performed via neuroendoscopic techniques. Under such circumstances, the specimens are already subcentimetre in size. After accounting for tissue prioritised for histopathological diagnosis, any remnant excess for further studies becomes even more limited. Nonetheless, this is the first in-depth molecular study of a mixed ethnic population of paediatric IGs from a uniquely Southeast Asian cohort. Highlights of this study includes profiling of tissue and corresponding CSF in the same patient via different genomic techniques. We observe findings that are both new and previously being published. Although our results are preliminary, they offer proof-of-concept for continued work in a larger cohort of patients to assess the relevance of novel findings in our study. A key consideration is to include germline testing as part of our study. This is because recent insights have highlighted novel germline variants in the gene *JMJD1C* in some overseas patients, and the existence of such remains unknown in our local cohort [12]. In meantime, a prospective, longitudinal study that includes the collection of blood as part of each patient’s biomaterial is already in place. Moving forward, efforts to include specific miRNAs and ctDNAs as part of a multimodality, targeted panel for individuals is certainly in the horizon. Putting it all together, in this era of targeted therapy, being cognizant of combining knowledge of genetic disposition and disease-driving mechanisms to tailor selective drugs should be a priority for affected patients.

## Conclusion

In summary, the authors describe an exploratory study in a selected group of multi-ethnic paediatric IG patients. Our findings add to the growing body of literature for this challenging brain tumour. Most importantly, international collaborations with like-minded researchers is paramount for better disease understanding and holistic patient care, especially in Southeast Asia.

## Supplementary Information

**Additional file 1: Supplementary Data A.** miRDB search result for miR-221-3p.

**Additional file 2: Supplementary Data B.** TargetScan 7.2 search result for miR-221-3p.

## Data Availability

All relevant data are within the paper. Additional data is available in the Supporting Information files.

## Abbreviations

CNS: Central nervous system
CSF: Cerebrospinal fluid
IG: Intracranial germinomas
miRNA: Micro-ribonuclease acid
NGS: Next-generation sequencing
PCR: Polymerase chain reaction
PI3K: Phosphoinositide 3-kinase
MAPK: Mitogen-activated protein kinase
WGS: Whole genome sequencing
αFP: Alpha-fetoprotein
βhCG: Beta-human chorionic gonadotropin

## References

[1] Sato K, Takeuchi H, Kubota T (2009). Pathology of intracranial germ cell tumors. Progress in neurological surgery.

[2] Aker FV, Berkman ZM, Aydingoz I, Hakan T, Toksoy G (2005). Pineal germinoma associated with multiple congenital melanocytic nevi: a unique presentation. Neuropathology..

[3] Report of Brain Tumor Registry of Japan (1984-2000). Neurol Med Chir (Tokyo). 2009;49 Suppl:PS1-96. PMID: 23914398. 23914398

[4] Nomura K (2001). Epidemiology of germ cell tumors in Asia of pineal region tumor. J Neuro-Oncol.

[5] Jennings MT, Gelman R, Hochberg F (1985). Intracranial germ-cell tumors: natural history and pathogenesis. J Neurosurg.

[6] Louis DN, Perry A, Reifenberger G, von Deimling A, Figarella-Branger D, Cavenee WK (2016). The 2016 World Health Organization classification of tumors of the central nervous system: a summary. Acta Neuropathol.

[7] Lin IJ, Shu SG, Chu HY, Chi CS (1997). Primary intracranial germ-cell tumor in children. Zhonghua Yi Xue Za Zhi (Taipei).

[8] Matsutani M, Sano K, Takakura K, Fujimaki T, Nakamura O, Funata N (1997). Primary intracranial germ cell tumors: a clinical analysis of 153 histologically verified cases. J Neurosurg.

[9] Huang X, Zhang R, Mao Y, Zhou LF, Zhang C (2016). Recent advances in molecular biology and treatment strategies for intracranial germ cell tumors. World J Pediatr.

[10] Yarden Y, Kuang WJ, Yang-Feng T, Coussens L, Munemitsu S, Dull TJ (1987). Human proto-oncogene c-kit: a new cell surface receptor tyrosine kinase for an unidentified ligand. EMBO J.

[11] Murray MJ, Nicholson JC, Coleman N (2015). Biology of childhood germ cell tumours, focussing on the significance of microRNAs. Andrology..

[12] Wang L, Yamaguchi S, Burstein MD, Terashima K, Chang K, Ng HK (2014). Novel somatic and germline mutations in intracranial germ cell tumours. Nature..

[13] Murray MJ, Bell E, Raby KL, Rijlaarsdam MA, Gillis AJ, Looijenga LH (2016). A pipeline to quantify serum and cerebrospinal fluid microRNAs for diagnosis and detection of relapse in paediatric malignant germ-cell tumours. Br J Cancer.

[14] Matsutani M (2001). Japanese pediatric brain tumor study G. combined chemotherapy and radiation therapy for CNS germ cell tumors--the Japanese experience. J Neuro-Oncol.

[15] Chen YW, Huang PI, Hu YW, Ho DM, Chang KP, Guo WY (2012). Treatment strategies for initially disseminated intracranial germinomas: experiences at a single institute. Childs Nerv Syst.

[16] Phua J, Kee AC, Tan A, Mukhopadhyay A, See KC, Aung NW (2011). End-of-life care in the general wards of a Singaporean hospital: an Asian perspective. J Palliat Med.

[17] Population in Brief 2014 2014 [Available from: http://www.nptd.gov.sg/portals/0/news/population-in-brief-2014.pdf.

[18] Wong LL, Zou R, Zhou L, Lim JY, Phua DCY, Liu C (2019). Combining circulating MicroRNA and NT-proBNP to detect and categorize heart failure subtypes. J Am Coll Cardiol.

[19] Saw WY, Tantoso E, Begum H, Zhou L, Zou R, He C (2017). Establishing multiple omics baselines for three southeast Asian populations in the Singapore integrative Omics study. Nat Commun.

[20] Ying L, Du L, Zou R, Shi L, Zhang N, Jin J (2020). Development of a serum miRNA panel for detection of early stage non-small cell lung cancer. Proc Natl Acad Sci U S A.

[21] Benjamini Y, Drai D, Elmer G, Kafkafi N, Golani I (2001). Controlling the false discovery rate in behavior genetics research. Behav Brain Res.

[22] Benjamini Y, Yekutieli D (2001). The control of the false discovery rate in multiple testing under dependency. Ann Stat.

[23] Wang HW, Wu YH, Hsieh JY, Liang ML, Chao ME, Liu DJ (2010). Pediatric primary central nervous system germ cell tumors of different prognosis groups show characteristic miRNome traits and chromosome copy number variations. BMC Genomics.

[24] Spiegel AM, Di Chiro G, Gorden P, Ommaya AK, Kolins J, Pomeroy TC (1976). Diagnosis of radiosensitive hypothalamic tumors without craniotomy: endocrine and neuroradiologic studies of intracranial atypical teratomas. Ann Intern Med.

[25] Inoue Y, Takeuchi T, Tamaki M, Nin K, Hakuba A, Nishimura S (1979). Sequential CT observations of irradiated intracranial germinomas. AJR Am J Roentgenol.

[26] Baumgartner JE, Edwards MS (1992). Pineal tumors. Neurosurg Clin N Am.

[27] Ono M, Ono M, Rhoton AL, Barry M (1984). Microsurgical anatomy of the region of the tentorial incisura. J Neurosurg.

[28] Shinoda J, Sakai N, Yano H, Hattori T, Ohkuma A, Sakaguchi H (2004). Prognostic factors and therapeutic problems of primary intracranial choriocarcinoma/germ-cell tumors with high levels of HCG. J Neuro-Oncol.

[29] Vaquero J, Martinez R, Manrique M (2000). Stereotactic biopsy for brain tumors: is it always necessary?. Surg Neurol.

[30] Lindsay H, Huang Y, Du Y, Braun FK, Teo WY, Kogiso M (2016). Preservation of KIT genotype in a novel pair of patient-derived orthotopic xenograft mouse models of metastatic pediatric CNS germinoma. J Neuro-Oncol.

[31] Kim JY, Park J (2015). Understanding the treatment strategies of intracranial germ cell tumors: focusing on radiotherapy. J Korean Neurosurg Soc.

[32] Packer RJ, Cohen BH, Cooney K (2000). Intracranial germ cell tumors. Oncologist.

[33] Poynter JN, Fonstad R, Tolar J, Spector LG, Ross JA (2014). Incidence of intracranial germ cell tumors by race in the United States, 1992-2010. J Neuro-Oncol.

[34] Bhoo-Pathy N, Hartman M, Yip CH, Saxena N, Taib NA, Lim SE (2012). Ethnic differences in survival after breast cancer in South East Asia. PLoS One.

[35] Teo MC, Soo KC (2013). Cancer trends and incidences in Singapore. Jpn J Clin Oncol.

[36] Wang H, Seow A, Lee HP (2004). Trends in cancer incidence among Singapore Malays: a low-risk population. Ann Acad Med Singap.

[37] Cheo ST, Lim GH, Lim KH (2017). Glioblastoma multiforme outcomes of 107 patients treated in two Singapore institutions. Singap Med J.

[38] Chiang PP, Lamoureux EL, Cheung CY, Sabanayagam C, Wong W, Tai ES (2011). Racial differences in the prevalence of diabetes but not diabetic retinopathy in a multi-ethnic Asian population. Invest Ophthalmol Vis Sci.

[39] Kurtz JE, Asmane I, Voegeli AC, Neuville A, Dufresne A, Litique V (2010). A V530I mutation in c-KIT exon 10 is associated to Imatinib response in Extraabdominal aggressive Fibromatosis. Sarcoma..

[40] Yim E, An HJ, Cho U, Kim Y, Kim SH, Choi YG (2018). Two different KIT mutations may lead to different responses to imatinib in metastatic gastrointestinal stromal tumor. Korean J Intern Med.

[41] Strohmeyer T, Reese D, Press M, Ackermann R, Hartmann M, Slamon D (1995). Expression of the c-kit proto-oncogene and its ligand stem cell factor (SCF) in normal and malignant human testicular tissue. J Urol.

[42] Goddard NC, McIntyre A, Summersgill B, Gilbert D, Kitazawa S, Shipley J (2007). KIT and RAS signalling pathways in testicular germ cell tumours: new data and a review of the literature. Int J Androl.

[43] Ichimura K, Fukushima S, Totoki Y, Matsushita Y, Otsuka A, Tomiyama A (2016). Recurrent neomorphic mutations of MTOR in central nervous system and testicular germ cell tumors may be targeted for therapy. Acta Neuropathol.

[44] Fukushima S, Otsuka A, Suzuki T, Yanagisawa T, Mishima K, Mukasa A (2014). Mutually exclusive mutations of KIT and RAS are associated with KIT mRNA expression and chromosomal instability in primary intracranial pure germinomas. Acta Neuropathol.

[45] Kamakura Y, Hasegawa M, Minamoto T, Yamashita J, Fujisawa H (2006). C-kit gene mutation: common and widely distributed in intracranial germinomas. J Neurosurg.

[46] Sakuma Y, Sakurai S, Oguni S, Satoh M, Hironaka M, Saito K (2004). C-kit gene mutations in intracranial germinomas. Cancer Sci.

[47] Bruno A, Boisselier B, Labreche K, Marie Y, Polivka M, Jouvet A (2014). Mutational analysis of primary central nervous system lymphoma. Oncotarget..

[48] Lee JY, Park K, Lim SH, Kim HS, Yoo KH, Jung KS (2015). Mutational profiling of brain metastasis from breast cancer: matched pair analysis of targeted sequencing between brain metastasis and primary breast cancer. Oncotarget..

[49] Wang Y, Springer S, Zhang M, McMahon KW, Kinde I, Dobbyn L (2015). Detection of tumor-derived DNA in cerebrospinal fluid of patients with primary tumors of the brain and spinal cord. Proc Natl Acad Sci U S A.

[50] Seoane J, De Mattos-Arruda L, Le Rhun E, Bardelli A, Weller M (2019). Cerebrospinal fluid cell-free tumour DNA as a liquid biopsy for primary brain tumours and central nervous system metastases. Ann Oncol.

[51] Xiao F, Lv S, Zong Z, Wu L, Tang X, Kuang W (2020). Cerebrospinal fluid biomarkers for brain tumor detection: clinical roles and current progress. Am J Transl Res.

[52] McEwen AE, Leary SES, Lockwood CM (2020). Beyond the blood: CSF-derived cfDNA for diagnosis and characterization of CNS tumors. Front Cell Dev Biol.

[53] Watanabe M, Kawaguchi T, Isa S, Ando M, Tamiya A, Kubo A (2015). Ultra-sensitive detection of the pretreatment EGFR T790M mutation in non-small cell lung Cancer patients with an EGFR-activating mutation using droplet digital PCR. Clin Cancer Res.

[54] Lynam-Lennon N, Maher SG, Reynolds JV (2009). The roles of microRNA in cancer and apoptosis. Biol Rev Camb Philos Soc.

[55] Croce CM (2009). Causes and consequences of microRNA dysregulation in cancer. Nat Rev Genet.

[56] Gonzalez-Gomez P, Sanchez P, Mira H (2011). MicroRNAs as regulators of neural stem cell-related pathways in glioblastoma multiforme. Mol Neurobiol.

[57] Bartel DP (2004). MicroRNAs: genomics, biogenesis, mechanism, and function. Cell..

[58] Yang W, Lee DY, Ben-David Y (2011). The roles of microRNAs in tumorigenesis and angiogenesis. Int J Physiol Pathophysiol Pharmacol.

[59] Chen X, Ba Y, Ma L, Cai X, Yin Y, Wang K (2008). Characterization of microRNAs in serum: a novel class of biomarkers for diagnosis of cancer and other diseases. Cell Res.

[60] Adlakha YK, Saini N (2014). Brain microRNAs and insights into biological functions and therapeutic potential of brain enriched miRNA-128. Mol Cancer.

[61] Knutsen E, Fiskaa T, Ursvik A, Jorgensen TE, Perander M, Lund E (2013). Performance comparison of digital microRNA profiling technologies applied on human breast Cancer cell lines. PLoS One.

[62] Schmittgen TD, Lee EJ, Jiang J, Sarkar A, Yang L, Elton TS (2008). Real-time PCR quantification of precursor and mature microRNA. Methods..

[63] Kang K, Peng X, Luo J, Gou D (2012). Identification of circulating miRNA biomarkers based on global quantitative real-time PCR profiling. J Animal Sci Biotechnology.

[64] Wan G, Lim QE, Too HP (2010). High-performance quantification of mature microRNAs by real-time RT-PCR using deoxyuridine-incorporated oligonucleotides and hemi-nested primers. Rna..

