# Supplementary material for: Molecular exploration of paediatric intracranial germinomas from multi-ethnic Singapore
Source: BMC Neurol. 2020 Nov 14;20:415. doi: 10.1186/s12883-020-01981-0 (PMC7666528; doi:10.1186/s12883-020-01981-0)
Supplement: Supplementary file 1 — Additional file 1: Supplementary Data A. miRDB search result for miR-221-3p. [file 12883_2020_1981_MOESM1_ESM.pdf]

miRDB

There are 615 predicted targets for hsa-miR-221-3p in miRDB.

Target Detail	Target Rank	Target Score	miRNA Name	Gene Symbol	Gene Description
Details	1	100	hsa-miR-221-3p	RIMS3	regulating synaptic membrane exocytosis 3
Details	2	100	hsa-miR-221-3p	GABRA1	gamma-aminobutyric acid type A receptor alpha1 subunit
Details	3	99	hsa-miR-221-3p	CDKN1B	cyclin dependent kinase inhibitor 1B
Details	4	98	hsa-miR-221-3p	PAIP1	poly(A) binding protein interacting protein 1
Details	5	98	hsa-miR-221-3p	PANK3	pantothenate kinase 3
Details	6	98	hsa-miR-221-3p	TCF12	transcription factor 12
Details	7	98	hsa-miR-221-3p	HECTD2	HECT domain E3 ubiquitin protein ligase 2
Details	8	97	hsa-miR-221-3p	RFX7	regulatory factor X7
Details	9	97	hsa-miR-221-3p	RGS6	regulator of G protein signaling 6
Details	10	97	hsa-miR-221-3p	HMBOX1	homeobox containing 1
Details	11	96	hsa-miR-221-3p	EIF5A2	eukaryotic translation initiation factor 5A2
Details	12	95	hsa-miR-221-3p	KIT	KIT proto-oncogene receptor tyrosine kinase
Details	13	95	hsa-miR-221-3p	MIDN	midnolin
Details	14	95	hsa-miR-221-3p	EML6	EMAP like 6
Details	15	95	hsa-miR-221-3p	CLVS2	clavesin 2
Details	16	95	hsa-miR-221-3p	GNAI3	G protein subunit alpha i3
Details	17	95	hsa-miR-221-3p	GRB10	growth factor receptor bound protein 10
Details	18	95	hsa-miR-221-3p	ARHGEF38	Rho guanine nucleotide exchange factor 38
Details	19	94	hsa-miR-221-3p	GRIK1	glutamate ionotropic receptor kainate type subunit 1
Details	20	94	hsa-miR-221-3p	TGS1	trimethylguanosine synthase 1
Details	21	94	hsa-miR-221-3p	RAB18	RAB18, member RAS oncogene family
Details	22	94	hsa-miR-221-3p	ADAM22	ADAM metallopeptidase domain 22
Details	23	94	hsa-miR-221-3p	DENND1B	DENN domain containing 1B
Details	24	94	hsa-miR-221-3p	FNIP2	folliculin interacting protein 2
Details	25	94	hsa-miR-221-3p	MYLIP	myosin regulatory light chain interacting protein
Details	26	94	hsa-miR-221-3p	VAPB	VAMP associated protein B and C
Details	27	94	hsa-miR-221-3p	FNDC3A	fibronectin type III domain containing 3A
Details	28	93	hsa-miR-221-3p	LOC100506388	uncharacterized LOC100506388
Details	29	93	hsa-miR-221-3p	ZNF91	zinc finger protein 91
Details	30	93	hsa-miR-221-3p	WDR35	WD repeat domain 35
Details	31	93	hsa-miR-221-3p	C3orf70	chromosome 3 open reading frame 70
Details	32	93	hsa-miR-221-3p	MIER3	MIER family member 3
Details	33	93	hsa-miR-221-3p	GALNT3	polypeptide N-acetylgalactosaminyltransferase 3
Details	34	93	hsa-miR-221-3p	NRK	Nik related kinase
Details	35	93	hsa-miR-221-3p	TFG	TRK-fused gene
Details	36	93	hsa-miR-221-3p	PCMTD1	protein-L-isoaspartate (D-aspartate) O-methyltransferase domain containing 1
Details	37	92	hsa-miR-221-3p	POGZ	pogo transposable element derived with

					ZNF domain
Details	38	92	hsa-miR-221-3p	RSBN1L	round spermatid basic protein 1 like
Details	39	92	hsa-miR-221-3p	DCUN1D1	defective in cullin neddylation 1 domain containing 1
Details	40	92	hsa-miR-221-3p	CHSY1	chondroitin sulfate synthase 1
Details	41	92	hsa-miR-221-3p	AGTPBP1	ATP/GTP binding protein 1
Details	42	92	hsa-miR-221-3p	SEC62	SEC62 homolog, preprotein translocation factor
Details	43	92	hsa-miR-221-3p	RIT2	Ras like without CAAX 2
Details	44	92	hsa-miR-221-3p	ERBB4	erb-b2 receptor tyrosine kinase 4
Details	45	92	hsa-miR-221-3p	DMRT3	doublesex and mab-3 related transcription factor 3
Details	46	92	hsa-miR-221-3p	PPP3R1	protein phosphatase 3 regulatory subunit B, alpha
Details	47	92	hsa-miR-221-3p	CCN1	cellular communication network factor 1
Details	48	92	hsa-miR-221-3p	NAA25	N(alpha)-acetyltransferase 25, NatB auxiliary subunit
Details	49	92	hsa-miR-221-3p	ARF4	ADP ribosylation factor 4
Details	50	92	hsa-miR-221-3p	CDH2	cadherin 2
Details	51	92	hsa-miR-221-3p	ZFPM2	zinc finger protein, FOG family member 2
Details	52	91	hsa-miR-221-3p	HNRNPH3	heterogeneous nuclear ribonucleoprotein H3
Details	53	91	hsa-miR-221-3p	KIF16B	kinesin family member 16B
Details	54	91	hsa-miR-221-3p	KIF20A	kinesin family member 20A
Details	55	91	hsa-miR-221-3p	FMR1	fragile X mental retardation 1
Details	56	91	hsa-miR-221-3p	ATXN1	ataxin 1
Details	57	91	hsa-miR-221-3p	FOXN2	forkhead box N2
Details	58	91	hsa-miR-221-3p	MRAP2	melanocortin 2 receptor accessory protein 2
Details	59	91	hsa-miR-221-3p	CBWD5	COBW domain containing 5
Details	60	91	hsa-miR-221-3p	CASZ1	castor zinc finger 1
Details	61	91	hsa-miR-221-3p	CBWD1	COBW domain containing 1
Details	62	91	hsa-miR-221-3p	SUN2	Sad1 and UNC84 domain containing 2
Details	63	91	hsa-miR-221-3p	CBWD6	COBW domain containing 6
Details	64	91	hsa-miR-221-3p	DCAF12	DDB1 and CUL4 associated factor 12
Details	65	91	hsa-miR-221-3p	VASH1	vasohibin 1
Details	66	91	hsa-miR-221-3p	TMCC1	transmembrane and coiled-coil domain family 1
Details	67	91	hsa-miR-221-3p	TP53BP2	tumor protein p53 binding protein 2
Details	68	91	hsa-miR-221-3p	CBWD3	COBW domain containing 3
Details	69	91	hsa-miR-221-3p	ETV3	ETS variant 3
Details	70	90	hsa-miR-221-3p	MARK1	microtubule affinity regulating kinase 1
Details	71	90	hsa-miR-221-3p	PHACTR4	phosphatase and actin regulator 4
Details	72	90	hsa-miR-221-3p	SYT10	synaptotagmin 10
Details	73	90	hsa-miR-221-3p	PIK3R1	phosphoinositide-3-kinase regulatory subunit 1
Details	74	90	hsa-miR-221-3p	CBWD2	COBW domain containing 2
Details	75	90	hsa-miR-221-3p	APOLD1	apolipoprotein L domain containing 1
Details	76	90	hsa-miR-221-3p	LHFPL2	LHFPL tetraspan subfamily member 2
Details	77	90	hsa-miR-221-3p	FERMT2	fermitin family member 2
Details	78	90	hsa-miR-221-3p	KLF7	Kruppel like factor 7
Details	79	89	hsa-miR-221-3p	CXCL12	C-X-C motif chemokine ligand 12
Details	80	89	hsa-miR-221-3p	CCDC18	coiled-coil domain containing 18
Details	81	89	hsa-miR-221-3p	NXPH1	neurexophilin 1
Details	82	89	hsa-miR-221-3p	BICDL1	BICD family like cargo adaptor 1
Details	83	89	hsa-miR-221-3p	BCL2L11	BCL2 like 11

Details	84	89	hsa-miR-221-3p	BRWD1	bromodomain and WD repeat domain containing 1
Details	85	89	hsa-miR-221-3p	C6	complement C6
Details	86	89	hsa-miR-221-3p	RFX8	RFX family member 8, lacking RFX DNA binding domain
Details	87	89	hsa-miR-221-3p	C6orf118	chromosome 6 open reading frame 118
Details	88	89	hsa-miR-221-3p	PPP2R2A	protein phosphatase 2 regulatory subunit Balpha
Details	89	89	hsa-miR-221-3p	MARF1	meiosis regulator and mRNA stability factor 1
Details	90	88	hsa-miR-221-3p	FAM214A	family with sequence similarity 214 member A
Details	91	88	hsa-miR-221-3p	PIEZO2	piezo type mechanosensitive ion channel component 2
Details	92	88	hsa-miR-221-3p	DPH6	diphthamine biosynthesis 6
Details	93	88	hsa-miR-221-3p	PLCL2	phospholipase C like 2
Details	94	88	hsa-miR-221-3p	ZNF615	zinc finger protein 615
Details	95	88	hsa-miR-221-3p	GABRG1	gamma-aminobutyric acid type A receptor gamma1 subunit
Details	96	88	hsa-miR-221-3p	CDK19	cyclin dependent kinase 19
Details	97	88	hsa-miR-221-3p	HIPK1	homeodomain interacting protein kinase 1
Details	98	88	hsa-miR-221-3p	GUCY1A2	guanylate cyclase 1 soluble subunit alpha 2
Details	99	88	hsa-miR-221-3p	WDR47	WD repeat domain 47
Details	100	87	hsa-miR-221-3p	SESN3	sestrin 3
Details	101	87	hsa-miR-221-3p	USP27X	ubiquitin specific peptidase 27 X-linked
Details	102	87	hsa-miR-221-3p	AP3B2	adaptor related protein complex 3 subunit beta 2
Details	103	87	hsa-miR-221-3p	ESR1	estrogen receptor 1
Details	104	87	hsa-miR-221-3p	TUB	tubby bipartite transcription factor
Details	105	87	hsa-miR-221-3p	ASPA	aspartoacylase
Details	106	87	hsa-miR-221-3p	SYBU	syntabulin
Details	107	87	hsa-miR-221-3p	CTDSPL2	CTD small phosphatase like 2
Details	108	87	hsa-miR-221-3p	SBK1	SH3 domain binding kinase 1
Details	109	86	hsa-miR-221-3p	DDIT4	DNA damage inducible transcript 4
Details	110	86	hsa-miR-221-3p	CLRN1	clarin 1
Details	111	86	hsa-miR-221-3p	SLC4A7	solute carrier family 4 member 7
Details	112	86	hsa-miR-221-3p	TLE3	TLE family member 3, transcriptional corepressor
Details	113	86	hsa-miR-221-3p	RFX3	regulatory factor X3
Details	114	86	hsa-miR-221-3p	EIF3J	eukaryotic translation initiation factor 3 subunit J
Details	115	86	hsa-miR-221-3p	ANKRD12	ankyrin repeat domain 12
Details	116	86	hsa-miR-221-3p	CREBL2	cAMP responsive element binding protein like 2
Details	117	86	hsa-miR-221-3p	TSPAN13	tetraspanin 13
Details	118	86	hsa-miR-221-3p	ZMYM2	zinc finger MYM-type containing 2
Details	119	86	hsa-miR-221-3p	DPP8	dipeptidyl peptidase 8
Details	120	86	hsa-miR-221-3p	AQP3	aquaporin 3 (Gill blood group)
Details	121	86	hsa-miR-221-3p	SHLD2	shieldin complex subunit 2
Details	122	85	hsa-miR-221-3p	MIA3	MIA SH3 domain ER export factor 3
Details	123	85	hsa-miR-221-3p	IRX5	iroquois homeobox 5
Details	124	85	hsa-miR-221-3p	CLGN	calmegin
Details	125	85	hsa-miR-221-3p	PGPEP1L	pyroglutamyl-peptidase I like
Details	126	85	hsa-miR-221-3p	KIAA1841	KIAA1841
Details	127	85	hsa-miR-221-3p	BEND4	BEN domain containing 4
Details	128	85	hsa-miR-221-3p	LYPLA1	lysophospholipase 1

Details	129	85	hsa-miR-221-3p	SEMA3C	semaphorin 3C
Details	130	85	hsa-miR-221-3p	PAF1	PAF1 homolog, Paf1/RNA polymerase II complex component
Details	131	85	hsa-miR-221-3p	TRPC3	transient receptor potential cation channel subfamily C member 3
Details	132	85	hsa-miR-221-3p	RNPS1	RNA binding protein with serine rich domain 1
Details	133	84	hsa-miR-221-3p	NIPAL4	NIPA like domain containing 4
Details	134	84	hsa-miR-221-3p	UBE2J1	ubiquitin conjugating enzyme E2 J1
Details	135	84	hsa-miR-221-3p	ZNF385A	zinc finger protein 385A
Details	136	84	hsa-miR-221-3p	TSC22D3	TSC22 domain family member 3
Details	137	84	hsa-miR-221-3p	ZNF181	zinc finger protein 181
Details	138	84	hsa-miR-221-3p	BEAN1	brain expressed associated with NEDD4 1
Details	139	84	hsa-miR-221-3p	KDR	kinase insert domain receptor
Details	140	84	hsa-miR-221-3p	GPBP1	GC-rich promoter binding protein 1
Details	141	84	hsa-miR-221-3p	SNX4	sorting nexin 4
Details	142	84	hsa-miR-221-3p	L3MBTL1	L3MBTL1, histone methyl-lysine binding protein
Details	143	84	hsa-miR-221-3p	AIDA	axin interactor, dorsalization associated
Details	144	84	hsa-miR-221-3p	GDF9	growth differentiation factor 9
Details	145	84	hsa-miR-221-3p	FGF14	fibroblast growth factor 14
Details	146	84	hsa-miR-221-3p	NYAP2	neuronal tyrosine-phosphorylated phosphoinositide-3-kinase adaptor 2
Details	147	83	hsa-miR-221-3p	MYBL1	MYB proto-oncogene like 1
Details	148	83	hsa-miR-221-3p	BRWD3	bromodomain and WD repeat domain containing 3
Details	149	83	hsa-miR-221-3p	POLR3E	RNA polymerase III subunit E
Details	150	83	hsa-miR-221-3p	IRF2	interferon regulatory factor 2
Details	151	83	hsa-miR-221-3p	ATP1B1	ATPase Na+/K+ transporting subunit beta 1
Details	152	83	hsa-miR-221-3p	TUBA1A	tubulin alpha 1a
Details	153	83	hsa-miR-221-3p	SNRNP48	small nuclear ribonucleoprotein U11/U12 subunit 48
Details	154	83	hsa-miR-221-3p	RNF4	ring finger protein 4
Details	155	83	hsa-miR-221-3p	DNAJC6	DnaJ heat shock protein family (Hsp40) member C6
Details	156	83	hsa-miR-221-3p	FXN	frataxin
Details	157	82	hsa-miR-221-3p	NAP1L5	nucleosome assembly protein 1 like 5
Details	158	82	hsa-miR-221-3p	BBC3	BCL2 binding component 3
Details	159	82	hsa-miR-221-3p	WNK3	WNK lysine deficient protein kinase 3
Details	160	82	hsa-miR-221-3p	SLC2A13	solute carrier family 2 member 13
Details	161	82	hsa-miR-221-3p	PLXNC1	plexin C1
Details	162	82	hsa-miR-221-3p	LBR	lamin B receptor
Details	163	82	hsa-miR-221-3p	RDX	radixin
Details	164	82	hsa-miR-221-3p	SYNCRIP	synaptotagmin binding cytoplasmic RNA interacting protein
Details	165	81	hsa-miR-221-3p	TDRP	testis development related protein
Details	166	81	hsa-miR-221-3p	SNAP29	synaptosome associated protein 29
Details	167	81	hsa-miR-221-3p	ATAD2B	ATPase family, AAA domain containing 2B
Details	168	81	hsa-miR-221-3p	AKAP5	A-kinase anchoring protein 5
Details	169	81	hsa-miR-221-3p	RBP2	retinol binding protein 2
Details	170	81	hsa-miR-221-3p	INA	internexin neuronal intermediate filament protein alpha
Details	171	81	hsa-miR-221-3p	LUZP2	leucine zipper protein 2
Details	172	81	hsa-miR-221-3p	SMARCA5	SWI/SNF related, matrix associated, actin dependent regulator of chromatin, subfamily a, member 5

Details	173	81	hsa-miR-221-3p	SVIP	small VCP interacting protein
Details	174	80	hsa-miR-221-3p	ZFYVE16	zinc finger FYVE-type containing 16
Details	175	80	hsa-miR-221-3p	PMEPA1	prostate transmembrane protein, androgen induced 1
Details	176	80	hsa-miR-221-3p	ZFP36L2	ZFP36 ring finger protein like 2
Details	177	80	hsa-miR-221-3p	ZNF624	zinc finger protein 624
Details	178	80	hsa-miR-221-3p	SEC24C	SEC24 homolog C, COPII coat complex component
Details	179	80	hsa-miR-221-3p	RALA	RAS like proto-oncogene A
Details	180	80	hsa-miR-221-3p	SLC6A4	solute carrier family 6 member 4
Details	181	80	hsa-miR-221-3p	RAB1A	RAB1A, member RAS oncogene family
Details	182	80	hsa-miR-221-3p	TNRC6C	trinucleotide repeat containing 6C
Details	183	80	hsa-miR-221-3p	HSPA8	heat shock protein family A (Hsp70) member 8
Details	184	80	hsa-miR-221-3p	CASR	calcium sensing receptor
Details	185	80	hsa-miR-221-3p	PRRC2B	proline rich coiled-coil 2B
Details	186	79	hsa-miR-221-3p	NFYB	nuclear transcription factor Y subunit beta
Details	187	79	hsa-miR-221-3p	NTF3	neurotrophin 3
Details	188	79	hsa-miR-221-3p	CMTM4	CKLF like MARVEL transmembrane domain containing 4
Details	189	79	hsa-miR-221-3p	PARP9	poly(ADP-ribose) polymerase family member 9
Details	190	79	hsa-miR-221-3p	NANOS1	nanos C2HC-type zinc finger 1
Details	191	79	hsa-miR-221-3p	ANGPTL2	angiopoietin like 2
Details	192	79	hsa-miR-221-3p	AMMECR1	Alport syndrome, mental retardation, midface hypoplasia and elliptocytosis chromosomal region gene 1
Details	193	79	hsa-miR-221-3p	OSTM1	osteoclastogenesis associated transmembrane protein 1
Details	194	78	hsa-miR-221-3p	SOX10	SRY-box 10
Details	195	78	hsa-miR-221-3p	DIRAS3	DIRAS family GTPase 3
Details	196	78	hsa-miR-221-3p	ADAM17	ADAM metallopeptidase domain 17
Details	197	78	hsa-miR-221-3p	LRFN2	leucine rich repeat and fibronectin type III domain containing 2
Details	198	78	hsa-miR-221-3p	ZNF275	zinc finger protein 275
Details	199	78	hsa-miR-221-3p	NFATC3	nuclear factor of activated T cells 3
Details	200	78	hsa-miR-221-3p	PTPN4	protein tyrosine phosphatase, non-receptor type 4
Details	201	78	hsa-miR-221-3p	ANKRD52	ankyrin repeat domain 52
Details	202	78	hsa-miR-221-3p	NFATC2	nuclear factor of activated T cells 2
Details	203	77	hsa-miR-221-3p	MAP4K5	mitogen-activated protein kinase kinase kinase kinase 5
Details	204	77	hsa-miR-221-3p	KMT2A	lysine methyltransferase 2A
Details	205	77	hsa-miR-221-3p	PLCXD3	phosphatidylinositol specific phospholipase C X domain containing 3
Details	206	77	hsa-miR-221-3p	ZFAND5	zinc finger AN1-type containing 5
Details	207	77	hsa-miR-221-3p	KCNH8	potassium voltage-gated channel subfamily H member 8
Details	208	77	hsa-miR-221-3p	NDST3	N-deacetylase and N-sulfotransferase 3
Details	209	77	hsa-miR-221-3p	SLC30A6	solute carrier family 30 member 6
Details	210	77	hsa-miR-221-3p	NCKAP5	NCK associated protein 5
Details	211	77	hsa-miR-221-3p	ANXA3	annexin A3
Details	212	77	hsa-miR-221-3p	EOGT	EGF domain specific O-linked N-acetylglucosamine transferase
Details	213	77	hsa-miR-221-3p	ZBTB5	zinc finger and BTB domain containing 5
Details	214	77	hsa-miR-221-3p	BMF	Bcl2 modifying factor
Details	215	77	hsa-miR-221-3p	CUX2	cut like homeobox 2

Details	216	76	hsa-miR-221-3p	PRDM1	PR/SET domain 1
Details	217	76	hsa-miR-221-3p	FRY	FRY microtubule binding protein
Details	218	76	hsa-miR-221-3p	CLDN11	claudin 11
Details	219	76	hsa-miR-221-3p	PLPPR1	phospholipid phosphatase related 1
Details	220	76	hsa-miR-221-3p	PPDPFL	pancreatic progenitor cell differentiation and proliferation factor like
Details	221	76	hsa-miR-221-3p	CNR1	cannabinoid receptor 1
Details	222	76	hsa-miR-221-3p	TRAF3IP2	TRAF3 interacting protein 2
Details	223	76	hsa-miR-221-3p	ITGB3	integrin subunit beta 3
Details	224	76	hsa-miR-221-3p	ZNF629	zinc finger protein 629
Details	225	76	hsa-miR-221-3p	ZFP30	ZFP30 zinc finger protein
Details	226	76	hsa-miR-221-3p	SNCB	synuclein beta
Details	227	76	hsa-miR-221-3p	CPEB3	cytoplasmic polyadenylation element binding protein 3
Details	228	76	hsa-miR-221-3p	GALNT18	polypeptide N-acetylgalactosaminyltransferase 18
Details	229	76	hsa-miR-221-3p	PRICKLE2	prickle planar cell polarity protein 2
Details	230	76	hsa-miR-221-3p	MEGF9	multiple EGF like domains 9
Details	231	75	hsa-miR-221-3p	PAIP2	poly(A) binding protein interacting protein 2
Details	232	75	hsa-miR-221-3p	PCDHA1	protocadherin alpha 1
Details	233	75	hsa-miR-221-3p	CBFB	core-binding factor subunit beta
Details	234	75	hsa-miR-221-3p	C11orf87	chromosome 11 open reading frame 87
Details	235	75	hsa-miR-221-3p	PCDHA4	protocadherin alpha 4
Details	236	75	hsa-miR-221-3p	PCDHA7	protocadherin alpha 7
Details	237	75	hsa-miR-221-3p	PCDHA3	protocadherin alpha 3
Details	238	75	hsa-miR-221-3p	PCDHA12	protocadherin alpha 12
Details	239	75	hsa-miR-221-3p	GBX2	gastrulation brain homeobox 2
Details	240	75	hsa-miR-221-3p	ZNF704	zinc finger protein 704
Details	241	75	hsa-miR-221-3p	C6orf120	chromosome 6 open reading frame 120
Details	242	75	hsa-miR-221-3p	PCDHA11	protocadherin alpha 11
Details	243	75	hsa-miR-221-3p	PCDHA2	protocadherin alpha 2
Details	244	75	hsa-miR-221-3p	DCUN1D4	defective in cullin neddylation 1 domain containing 4
Details	245	75	hsa-miR-221-3p	FBXO47	F-box protein 47
Details	246	75	hsa-miR-221-3p	XIRP2	xin actin binding repeat containing 2
Details	247	75	hsa-miR-221-3p	PCDHA6	protocadherin alpha 6
Details	248	75	hsa-miR-221-3p	MAGI1	membrane associated guanylate kinase, WW and PDZ domain containing 1
Details	249	75	hsa-miR-221-3p	PCDHA5	protocadherin alpha 5
Details	250	75	hsa-miR-221-3p	ZKSCAN8	zinc finger with KRAB and SCAN domains 8
Details	251	75	hsa-miR-221-3p	PCDHA10	protocadherin alpha 10
Details	252	75	hsa-miR-221-3p	PCDHAC1	protocadherin alpha subfamily C, 1
Details	253	75	hsa-miR-221-3p	PCDHA8	protocadherin alpha 8
Details	254	75	hsa-miR-221-3p	PCDHA13	protocadherin alpha 13
Details	255	75	hsa-miR-221-3p	PCDHAC2	protocadherin alpha subfamily C, 2
Details	256	75	hsa-miR-221-3p	CPNE8	copine 8
Details	257	75	hsa-miR-221-3p	PPARGC1A	PPARG coactivator 1 alpha
Details	258	75	hsa-miR-221-3p	TMEM132C	transmembrane protein 132C
Details	259	74	hsa-miR-221-3p	ABHD3	abhydrolase domain containing 3
Details	260	74	hsa-miR-221-3p	MPZL1	myelin protein zero like 1
Details	261	74	hsa-miR-221-3p	VGLL4	vestigial like family member 4
Details	262	74	hsa-miR-221-3p	SRD5A3	steroid 5 alpha-reductase 3
Details	263	74	hsa-miR-221-3p	RORB	RAR related orphan receptor B
Details	264	74	hsa-miR-221-3p	SCD5	stearoyl-CoA desaturase 5

Details	265	74	hsa-miR-221-3p	RALGAPA1	Ral GTPase activating protein catalytic alpha subunit 1
Details	266	73	hsa-miR-221-3p	MRPS7	mitochondrial ribosomal protein S7
Details	267	73	hsa-miR-221-3p	FANCD2	FA complementation group D2
Details	268	73	hsa-miR-221-3p	CLDND1	claudin domain containing 1
Details	269	73	hsa-miR-221-3p	SYCE2	synaptonemal complex central element protein 2
Details	270	73	hsa-miR-221-3p	YWHAG	tyrosine 3-monooxygenase/tryptophan 5-monooxygenase activation protein gamma
Details	271	73	hsa-miR-221-3p	TGOLN2	trans-golgi network protein 2
Details	272	73	hsa-miR-221-3p	KLC1	kinesin light chain 1
Details	273	73	hsa-miR-221-3p	ABCA8	ATP binding cassette subfamily A member 8
Details	274	73	hsa-miR-221-3p	ZNF93	zinc finger protein 93
Details	275	73	hsa-miR-221-3p	SGIP1	SH3 domain GRB2 like endophilin interacting protein 1
Details	276	73	hsa-miR-221-3p	PDIK1L	PDLIM1 interacting kinase 1 like
Details	277	73	hsa-miR-221-3p	ARAP2	ArfGAP with RhoGAP domain, ankyrin repeat and PH domain 2
Details	278	73	hsa-miR-221-3p	WSB2	WD repeat and SOCS box containing 2
Details	279	73	hsa-miR-221-3p	SPART	spartin
Details	280	73	hsa-miR-221-3p	PTBP3	polypyrimidine tract binding protein 3
Details	281	72	hsa-miR-221-3p	NIPBL	NIPBL, cohesin loading factor
Details	282	72	hsa-miR-221-3p	NOVA1	NOVA alternative splicing regulator 1
Details	283	72	hsa-miR-221-3p	ZNF74	zinc finger protein 74
Details	284	72	hsa-miR-221-3p	GAB1	GRB2 associated binding protein 1
Details	285	72	hsa-miR-221-3p	GTF2E1	general transcription factor IIE subunit 1
Details	286	72	hsa-miR-221-3p	CAMTA1	calmodulin binding transcription activator 1
Details	287	72	hsa-miR-221-3p	ONECUT2	one cut homeobox 2
Details	288	72	hsa-miR-221-3p	PLSCR4	phospholipid scramblase 4
Details	289	72	hsa-miR-221-3p	SERPINB2	serpin family B member 2
Details	290	72	hsa-miR-221-3p	EVI2A	ecotropic viral integration site 2A
Details	291	72	hsa-miR-221-3p	PDS5A	PDS5 cohesin associated factor A
Details	292	72	hsa-miR-221-3p	TLNRD1	talin rod domain containing 1
Details	293	72	hsa-miR-221-3p	DYNC1LI2	dynein cytoplasmic 1 light intermediate chain 2
Details	294	72	hsa-miR-221-3p	MAN2A1	mannosidase alpha class 2A member 1
Details	295	72	hsa-miR-221-3p	AGPS	alkylglycerone phosphate synthase
Details	296	72	hsa-miR-221-3p	TBXT	T-box transcription factor T
Details	297	72	hsa-miR-221-3p	DBT	dihydrolipoamide branched chain transacylase E2
Details	298	71	hsa-miR-221-3p	ENAH	ENAH, actin regulator
Details	299	71	hsa-miR-221-3p	LRRCC1	leucine rich repeat and coiled-coil centrosomal protein 1
Details	300	71	hsa-miR-221-3p	CREBZF	CREB/ATF bZIP transcription factor
Details	301	71	hsa-miR-221-3p	CDKN2AIP	CDKN2A interacting protein
Details	302	71	hsa-miR-221-3p	CYLD	CYLD lysine 63 deubiquitinase
Details	303	71	hsa-miR-221-3p	RBM24	RNA binding motif protein 24
Details	304	71	hsa-miR-221-3p	KIAA0586	KIAA0586
Details	305	71	hsa-miR-221-3p	ARHGEF7	Rho guanine nucleotide exchange factor 7
Details	306	71	hsa-miR-221-3p	ITPR2	inositol 1,4,5-trisphosphate receptor type 2
Details	307	71	hsa-miR-221-3p	KPNA2	karyopherin subunit alpha 2
Details	308	71	hsa-miR-221-3p	ZNF25	zinc finger protein 25
Details	309	71	hsa-miR-221-3p	PDLIM2	PDZ and LIM domain 2

Details	310	70	hsa-miR-221-3p	TOX	thymocyte selection associated high mobility group box
Details	311	70	hsa-miR-221-3p	PRKAB2	protein kinase AMP-activated non-catalytic subunit beta 2
Details	312	70	hsa-miR-221-3p	NSMCE4A	NSE4 homolog A, SMC5-SMC6 complex component
Details	313	70	hsa-miR-221-3p	SEC24B	SEC24 homolog B, COPII coat complex component
Details	314	70	hsa-miR-221-3p	TRPS1	transcriptional repressor GATA binding 1
Details	315	70	hsa-miR-221-3p	CLIC2	chloride intracellular channel 2
Details	316	70	hsa-miR-221-3p	NAP1L1	nucleosome assembly protein 1 like 1
Details	317	70	hsa-miR-221-3p	LRRTM2	leucine rich repeat transmembrane neuronal 2
Details	318	70	hsa-miR-221-3p	IL17RB	interleukin 17 receptor B
Details	319	70	hsa-miR-221-3p	SEMA6D	semaphorin 6D
Details	320	70	hsa-miR-221-3p	HNRNPA0	heterogeneous nuclear ribonucleoprotein A0
Details	321	70	hsa-miR-221-3p	GJC3	gap junction protein gamma 3
Details	322	70	hsa-miR-221-3p	CYREN	cell cycle regulator of NHEJ
Details	323	69	hsa-miR-221-3p	SOCS3	suppressor of cytokine signaling 3
Details	324	69	hsa-miR-221-3p	CYP4X1	cytochrome P450 family 4 subfamily X member 1
Details	325	69	hsa-miR-221-3p	KPNA1	karyopherin subunit alpha 1
Details	326	69	hsa-miR-221-3p	ANKRD10	ankyrin repeat domain 10
Details	327	69	hsa-miR-221-3p	NLK	nemo like kinase
Details	328	69	hsa-miR-221-3p	MEX3A	mex-3 RNA binding family member A
Details	329	69	hsa-miR-221-3p	HLTF	helicase like transcription factor
Details	330	69	hsa-miR-221-3p	IFIT2	interferon induced protein with tetratricopeptide repeats 2
Details	331	69	hsa-miR-221-3p	ANKS1B	ankyrin repeat and sterile alpha motif domain containing 1B
Details	332	69	hsa-miR-221-3p	UGT2B15	UDP glucuronosyltransferase family 2 member B15
Details	333	69	hsa-miR-221-3p	CHD7	chromodomain helicase DNA binding protein 7
Details	334	69	hsa-miR-221-3p	IGF2BP2	insulin like growth factor 2 mRNA binding protein 2
Details	335	69	hsa-miR-221-3p	YTHDF3	YTH N6-methyladenosine RNA binding protein 3
Details	336	68	hsa-miR-221-3p	NGRN	neugrin, neurite outgrowth associated
Details	337	68	hsa-miR-221-3p	COBLL1	cordon-bleu WH2 repeat protein like 1
Details	338	68	hsa-miR-221-3p	CEP41	centrosomal protein 41
Details	339	68	hsa-miR-221-3p	ASB4	ankyrin repeat and SOCS box containing 4
Details	340	68	hsa-miR-221-3p	PDCD10	programmed cell death 10
Details	341	68	hsa-miR-221-3p	MASTL	microtubule associated serine/threonine kinase like
Details	342	68	hsa-miR-221-3p	RUNX2	runt related transcription factor 2
Details	343	68	hsa-miR-221-3p	TRABD2B	TraB domain containing 2B
Details	344	68	hsa-miR-221-3p	PTPRZ1	protein tyrosine phosphatase, receptor type Z1
Details	345	68	hsa-miR-221-3p	FAT2	FAT atypical cadherin 2
Details	346	68	hsa-miR-221-3p	FAM160B1	family with sequence similarity 160 member B1
Details	347	68	hsa-miR-221-3p	KANK4	KN motif and ankyrin repeat domains 4
Details	348	68	hsa-miR-221-3p	ZNF652	zinc finger protein 652
Details	349	67	hsa-miR-221-3p	CD164	CD164 molecule
Details	350	67	hsa-miR-221-3p	NRG1	neuregulin 1

Details	351	67	hsa-miR-221-3p	GNB1	G protein subunit beta 1
Details	352	67	hsa-miR-221-3p	CEP70	centrosomal protein 70
Details	353	67	hsa-miR-221-3p	CCSAP	centriole, cilia and spindle associated protein
Details	354	67	hsa-miR-221-3p	KHDRBS2	KH RNA binding domain containing, signal transduction associated 2
Details	355	67	hsa-miR-221-3p	ERCC4	ERCC excision repair 4, endonuclease catalytic subunit
Details	356	67	hsa-miR-221-3p	ACVR2B	activin A receptor type 2B
Details	357	67	hsa-miR-221-3p	RASSF8	Ras association domain family member 8
Details	358	66	hsa-miR-221-3p	RNF20	ring finger protein 20
Details	359	66	hsa-miR-221-3p	ALDH16A1	aldehyde dehydrogenase 16 family member A1
Details	360	66	hsa-miR-221-3p	SPTBN1	spectrin beta, non-erythrocytic 1
Details	361	66	hsa-miR-221-3p	ANTXR2	ANTXR cell adhesion molecule 2
Details	362	66	hsa-miR-221-3p	ANKHD1	ankyrin repeat and KH domain containing 1
Details	363	66	hsa-miR-221-3p	SLC4A4	solute carrier family 4 member 4
Details	364	66	hsa-miR-221-3p	ZNF3	zinc finger protein 3
Details	365	66	hsa-miR-221-3p	CALM1	calmodulin 1
Details	366	66	hsa-miR-221-3p	BCL2L14	BCL2 like 14
Details	367	66	hsa-miR-221-3p	TYMSOS	TYMS opposite strand
Details	368	66	hsa-miR-221-3p	CARF	calcium responsive transcription factor
Details	369	66	hsa-miR-221-3p	USP6NL	USP6 N-terminal like
Details	370	66	hsa-miR-221-3p	ZC3H13	zinc finger CCCH-type containing 13
Details	371	66	hsa-miR-221-3p	DLD	dihydrolipoamide dehydrogenase
Details	372	66	hsa-miR-221-3p	FBXO28	F-box protein 28
Details	373	66	hsa-miR-221-3p	MON2	MON2 homolog, regulator of endosome-to-Golgi trafficking
Details	374	66	hsa-miR-221-3p	STN1	STN1, CST complex subunit
Details	375	66	hsa-miR-221-3p	ETS1	ETS proto-oncogene 1, transcription factor
Details	376	65	hsa-miR-221-3p	CACNB4	calcium voltage-gated channel auxiliary subunit beta 4
Details	377	65	hsa-miR-221-3p	PDCD6IP	programmed cell death 6 interacting protein
Details	378	65	hsa-miR-221-3p	GOLGA1	golgin A1
Details	379	65	hsa-miR-221-3p	ZFHX3	zinc finger homeobox 3
Details	380	65	hsa-miR-221-3p	TMEM132B	transmembrane protein 132B
Details	381	65	hsa-miR-221-3p	C22orf39	chromosome 22 open reading frame 39
Details	382	65	hsa-miR-221-3p	CCT5	chaperonin containing TCP1 subunit 5
Details	383	65	hsa-miR-221-3p	KSR1	kinase suppressor of ras 1
Details	384	65	hsa-miR-221-3p	NUFIP2	nuclear FMR1 interacting protein 2
Details	385	65	hsa-miR-221-3p	AGFG1	ArfGAP with FG repeats 1
Details	386	65	hsa-miR-221-3p	TCF7L2	transcription factor 7 like 2
Details	387	65	hsa-miR-221-3p	PPP4R2	protein phosphatase 4 regulatory subunit 2
Details	388	65	hsa-miR-221-3p	STK24	serine/threonine kinase 24
Details	389	65	hsa-miR-221-3p	CDON	cell adhesion associated, oncogene regulated
Details	390	65	hsa-miR-221-3p	FAM222B	family with sequence similarity 222 member B
Details	391	65	hsa-miR-221-3p	DGKH	diacylglycerol kinase eta
Details	392	64	hsa-miR-221-3p	HDLBP	high density lipoprotein binding protein
Details	393	64	hsa-miR-221-3p	SHANK2	SH3 and multiple ankyrin repeat domains 2
Details	394	64	hsa-miR-221-3p	CFAP161	cilia and flagella associated protein 161
Details	395	64	hsa-miR-221-3p	CRHBP	corticotropin releasing hormone binding

					protein
Details	396	64	hsa-miR-221-3p	CDV3	CDV3 homolog
Details	397	64	hsa-miR-221-3p	URI1	URI1, prefoldin like chaperone
Details	398	64	hsa-miR-221-3p	CD47	CD47 molecule
Details	399	64	hsa-miR-221-3p	SPPL3	signal peptide peptidase like 3
Details	400	64	hsa-miR-221-3p	CD4	CD4 molecule
Details	401	64	hsa-miR-221-3p	LRRC19	leucine rich repeat containing 19
Details	402	64	hsa-miR-221-3p	EMX2	empty spiracles homeobox 2
Details	403	64	hsa-miR-221-3p	PRDM11	PR/SET domain 11
Details	404	64	hsa-miR-221-3p	PTPRR	protein tyrosine phosphatase, receptor type R
Details	405	64	hsa-miR-221-3p	IGDCC4	immunoglobulin superfamily DCC subclass member 4
Details	406	64	hsa-miR-221-3p	CDKN2B	cyclin dependent kinase inhibitor 2B
Details	407	64	hsa-miR-221-3p	TMEM25	transmembrane protein 25
Details	408	64	hsa-miR-221-3p	CAMK1D	calcium/calmodulin dependent protein kinase ID
Details	409	63	hsa-miR-221-3p	MGAT4A	alpha-1,3-mannosyl-glycoprotein 4-beta-N-acetylglucosaminyltransferase A
Details	410	63	hsa-miR-221-3p	ZFP90	ZFP90 zinc finger protein
Details	411	63	hsa-miR-221-3p	ERMN	ermin
Details	412	63	hsa-miR-221-3p	RPS3	ribosomal protein S3
Details	413	63	hsa-miR-221-3p	CCP110	centriolar coiled-coil protein 110
Details	414	63	hsa-miR-221-3p	NBPF3	NBPF member 3
Details	415	63	hsa-miR-221-3p	CRKL	CRK like proto-oncogene, adaptor protein
Details	416	63	hsa-miR-221-3p	REV3L	REV3 like, DNA directed polymerase zeta catalytic subunit
Details	417	63	hsa-miR-221-3p	STYX	serine/threonine/tyrosine interacting protein
Details	418	63	hsa-miR-221-3p	ZNF83	zinc finger protein 83
Details	419	63	hsa-miR-221-3p	ST8SIA1	ST8 alpha-N-acetyl-neuraminide alpha-2,8-sialyltransferase 1
Details	420	63	hsa-miR-221-3p	SOD2	superoxide dismutase 2
Details	421	63	hsa-miR-221-3p	SEPT14	septin 14
Details	422	63	hsa-miR-221-3p	GPD2	glycerol-3-phosphate dehydrogenase 2
Details	423	63	hsa-miR-221-3p	HIPK2	homeodomain interacting protein kinase 2
Details	424	63	hsa-miR-221-3p	HOXC10	homeobox C10
Details	425	63	hsa-miR-221-3p	STOX2	storkhead box 2
Details	426	62	hsa-miR-221-3p	PSAP	prosaposin
Details	427	62	hsa-miR-221-3p	DIRAS2	DIRAS family GTPase 2
Details	428	62	hsa-miR-221-3p	C2CD4A	C2 calcium dependent domain containing 4A
Details	429	62	hsa-miR-221-3p	SLC26A3	solute carrier family 26 member 3
Details	430	62	hsa-miR-221-3p	DLG2	discs large MAGUK scaffold protein 2
Details	431	62	hsa-miR-221-3p	PRDM2	PR/SET domain 2
Details	432	62	hsa-miR-221-3p	C18orf54	chromosome 18 open reading frame 54
Details	433	62	hsa-miR-221-3p	CRX	cone-rod homeobox
Details	434	62	hsa-miR-221-3p	CAVIN3	caveolae associated protein 3
Details	435	62	hsa-miR-221-3p	STK38L	serine/threonine kinase 38 like
Details	436	62	hsa-miR-221-3p	CCDC126	coiled-coil domain containing 126
Details	437	62	hsa-miR-221-3p	GLMN	glomulin, FKBP associated protein
Details	438	62	hsa-miR-221-3p	LGI2	leucine rich repeat LGI family member 2
Details	439	62	hsa-miR-221-3p	KBTBD11	kelch repeat and BTB domain containing 11
Details	440	62	hsa-miR-221-3p	PLEKHA2	pleckstrin homology domain containing A2
Details	441	61	hsa-miR-221-3p	SLITRK5	SLIT and NTRK like family member 5

Details	442	61	hsa-miR-221-3p	THBS1	thrombospondin 1
Details	443	61	hsa-miR-221-3p	PCDHA9	protocadherin alpha 9
Details	444	61	hsa-miR-221-3p	GLS	glutaminase
Details	445	61	hsa-miR-221-3p	CCDC148	coiled-coil domain containing 148
Details	446	61	hsa-miR-221-3p	SLC10A7	solute carrier family 10 member 7
Details	447	61	hsa-miR-221-3p	MSL2	MSL complex subunit 2
Details	448	61	hsa-miR-221-3p	MBD2	methyl-CpG binding domain protein 2
Details	449	61	hsa-miR-221-3p	PLPP3	phospholipid phosphatase 3
Details	450	61	hsa-miR-221-3p	TMEM237	transmembrane protein 237
Details	451	61	hsa-miR-221-3p	PROM2	prominin 2
Details	452	61	hsa-miR-221-3p	UBE2V1	ubiquitin conjugating enzyme E2 V1
Details	453	61	hsa-miR-221-3p	MAPK10	mitogen-activated protein kinase 10
Details	454	61	hsa-miR-221-3p	ADIPOR1	adiponectin receptor 1
Details	455	61	hsa-miR-221-3p	TMEM167A	transmembrane protein 167A
Details	456	61	hsa-miR-221-3p	KCNQ3	potassium voltage-gated channel subfamily Q member 3
Details	457	60	hsa-miR-221-3p	LDHAL6B	lactate dehydrogenase A like 6B
Details	458	60	hsa-miR-221-3p	ANKIB1	ankyrin repeat and IBR domain containing 1
Details	459	60	hsa-miR-221-3p	ECPAS	Ecm29 proteasome adaptor and scaffold
Details	460	60	hsa-miR-221-3p	MRE11	MRE11 homolog, double strand break repair nuclease
Details	461	60	hsa-miR-221-3p	DCAF7	DDB1 and CUL4 associated factor 7
Details	462	60	hsa-miR-221-3p	ZNF547	zinc finger protein 547
Details	463	60	hsa-miR-221-3p	GRM1	glutamate metabotropic receptor 1
Details	464	60	hsa-miR-221-3p	GPM6A	glycoprotein M6A
Details	465	60	hsa-miR-221-3p	BRD1	bromodomain containing 1
Details	466	60	hsa-miR-221-3p	FBN2	fibrillin 2
Details	467	60	hsa-miR-221-3p	SLC25A37	solute carrier family 25 member 37
Details	468	60	hsa-miR-221-3p	TMSB15B	thymosin beta 15B
Details	469	60	hsa-miR-221-3p	CSTF2T	cleavage stimulation factor subunit 2 tau variant
Details	470	60	hsa-miR-221-3p	RECK	reversion inducing cysteine rich protein with kazal motifs
Details	471	60	hsa-miR-221-3p	TBC1D22B	TBC1 domain family member 22B
Details	472	60	hsa-miR-221-3p	SH3PXD2B	SH3 and PX domains 2B
Details	473	60	hsa-miR-221-3p	MYO10	myosin X
Details	474	59	hsa-miR-221-3p	ACADM	acyl-CoA dehydrogenase medium chain
Details	475	59	hsa-miR-221-3p	PRUNE1	prune exopolyphosphatase 1
Details	476	59	hsa-miR-221-3p	MRAP	melanocortin 2 receptor accessory protein
Details	477	59	hsa-miR-221-3p	PALM2	paralemmin 2
Details	478	59	hsa-miR-221-3p	IFRD1	interferon related developmental regulator 1
Details	479	59	hsa-miR-221-3p	CDK8	cyclin dependent kinase 8
Details	480	59	hsa-miR-221-3p	ACTC1	actin, alpha, cardiac muscle 1
Details	481	59	hsa-miR-221-3p	CNPY2	canopy FGF signaling regulator 2
Details	482	59	hsa-miR-221-3p	MAGEL2	MAGE family member L2
Details	483	58	hsa-miR-221-3p	TMEM165	transmembrane protein 165
Details	484	58	hsa-miR-221-3p	AADAC	arylacetamide deacetylase
Details	485	58	hsa-miR-221-3p	PLXDC2	plexin domain containing 2
Details	486	58	hsa-miR-221-3p	SKP1	S-phase kinase associated protein 1
Details	487	58	hsa-miR-221-3p	NDFIP1	Nedd4 family interacting protein 1
Details	488	58	hsa-miR-221-3p	CTTN	cortactin
Details	489	58	hsa-miR-221-3p	RETREG1	reticulophagy regulator 1
Details	490	58	hsa-miR-221-3p	CFHR5	complement factor H related 5
Details	491	58	hsa-miR-221-3p	HLA-F	major histocompatibility complex, class I,

					F
Details	492	58	hsa-miR-221-3p	C16orf45	chromosome 16 open reading frame 45
Details	493	58	hsa-miR-221-3p	NDUFA1	NADH:ubiquinone oxidoreductase subunit A1
Details	494	58	hsa-miR-221-3p	ENTPD7	ectonucleoside triphosphate diphosphohydrolase 7
Details	495	58	hsa-miR-221-3p	PTPRM	protein tyrosine phosphatase, receptor type M
Details	496	57	hsa-miR-221-3p	DEPDC4	DEP domain containing 4
Details	497	57	hsa-miR-221-3p	LRRN1	leucine rich repeat neuronal 1
Details	498	57	hsa-miR-221-3p	KCNK2	potassium two pore domain channel subfamily K member 2
Details	499	57	hsa-miR-221-3p	CTCF	CCCTC-binding factor
Details	500	57	hsa-miR-221-3p	KLF3	Kruppel like factor 3
Details	501	57	hsa-miR-221-3p	HTT	huntingtin
Details	502	57	hsa-miR-221-3p	HEXIM1	HEXIM P-TEFb complex subunit 1
Details	503	57	hsa-miR-221-3p	SATB1	SATB homeobox 1
Details	504	57	hsa-miR-221-3p	OLA1	Obg like ATPase 1
Details	505	57	hsa-miR-221-3p	SLC25A46	solute carrier family 25 member 46
Details	506	57	hsa-miR-221-3p	OSBPL3	oxysterol binding protein like 3
Details	507	57	hsa-miR-221-3p	IPO5	importin 5
Details	508	57	hsa-miR-221-3p	FKTN	fukutin
Details	509	57	hsa-miR-221-3p	SOX11	SRY-box 11
Details	510	57	hsa-miR-221-3p	SLC40A1	solute carrier family 40 member 1
Details	511	57	hsa-miR-221-3p	SPATS2L	spermatogenesis associated serine rich 2 like
Details	512	56	hsa-miR-221-3p	MAPK8	mitogen-activated protein kinase 8
Details	513	56	hsa-miR-221-3p	GALC	galactosylceramidase
Details	514	56	hsa-miR-221-3p	FIGNL2	fidgetin like 2
Details	515	56	hsa-miR-221-3p	RREB1	ras responsive element binding protein 1
Details	516	56	hsa-miR-221-3p	MCMDC2	minichromosome maintenance domain containing 2
Details	517	56	hsa-miR-221-3p	BBS4	Bardet-Biedl syndrome 4
Details	518	56	hsa-miR-221-3p	PGGT1B	protein geranylgeranyltransferase type I subunit beta
Details	519	56	hsa-miR-221-3p	FOS	Fos proto-oncogene, AP-1 transcription factor subunit
Details	520	56	hsa-miR-221-3p	TDRKH	tudor and KH domain containing
Details	521	56	hsa-miR-221-3p	PRKAA2	protein kinase AMP-activated catalytic subunit alpha 2
Details	522	56	hsa-miR-221-3p	SLITRK6	SLIT and NTRK like family member 6
Details	523	56	hsa-miR-221-3p	RBM18	RNA binding motif protein 18
Details	524	55	hsa-miR-221-3p	TAOK1	TAO kinase 1
Details	525	55	hsa-miR-221-3p	CXCL11	C-X-C motif chemokine ligand 11
Details	526	55	hsa-miR-221-3p	ETS2	ETS proto-oncogene 2, transcription factor
Details	527	55	hsa-miR-221-3p	COX15	cytochrome c oxidase assembly homolog COX15
Details	528	55	hsa-miR-221-3p	ATP2B3	ATPase plasma membrane Ca2+ transporting 3
Details	529	55	hsa-miR-221-3p	ARID1A	AT-rich interaction domain 1A
Details	530	55	hsa-miR-221-3p	RAB14	RAB14, member RAS oncogene family
Details	531	55	hsa-miR-221-3p	NAV3	neuron navigator 3
Details	532	55	hsa-miR-221-3p	ZNF595	zinc finger protein 595
Details	533	55	hsa-miR-221-3p	ASB7	ankyrin repeat and SOCS box containing 7
Details	534	55	hsa-miR-221-3p	ADHFE1	alcohol dehydrogenase, iron containing 1
Details	535	55	hsa-miR-221-3p	KBTBD8	kelch repeat and BTB domain containing 8

Details	536	55	hsa-miR-221-3p	PDZRN4	PDZ domain containing ring finger 4
Details	537	55	hsa-miR-221-3p	STMN1	stathmin 1
Details	538	55	hsa-miR-221-3p	PAFAH1B2	platelet activating factor acetylhydrolase 1b catalytic subunit 2
Details	539	55	hsa-miR-221-3p	CASP3	caspase 3
Details	540	55	hsa-miR-221-3p	MAT2A	methionine adenosyltransferase 2A
Details	541	55	hsa-miR-221-3p	BCHE	butyrylcholinesterase
Details	542	55	hsa-miR-221-3p	DDX42	DEAD-box helicase 42
Details	543	54	hsa-miR-221-3p	GTF2B	general transcription factor IIB
Details	544	54	hsa-miR-221-3p	PITPNM2	phosphatidylinositol transfer protein membrane associated 2
Details	545	54	hsa-miR-221-3p	KLHL18	kelch like family member 18
Details	546	54	hsa-miR-221-3p	RNF44	ring finger protein 44
Details	547	54	hsa-miR-221-3p	SLC45A3	solute carrier family 45 member 3
Details	548	54	hsa-miR-221-3p	SLC6A9	solute carrier family 6 member 9
Details	549	54	hsa-miR-221-3p	LIFR	LIF receptor alpha
Details	550	54	hsa-miR-221-3p	ARHGAP42	Rho GTPase activating protein 42
Details	551	54	hsa-miR-221-3p	VEZF1	vascular endothelial zinc finger 1
Details	552	54	hsa-miR-221-3p	RAB3GAP2	RAB3 GTPase activating non-catalytic protein subunit 2
Details	553	54	hsa-miR-221-3p	MTMR6	myotubularin related protein 6
Details	554	53	hsa-miR-221-3p	MED1	mediator complex subunit 1
Details	555	53	hsa-miR-221-3p	COL6A5	collagen type VI alpha 5 chain
Details	556	53	hsa-miR-221-3p	USP49	ubiquitin specific peptidase 49
Details	557	53	hsa-miR-221-3p	RGS8	regulator of G protein signaling 8
Details	558	53	hsa-miR-221-3p	CD8A	CD8a molecule
Details	559	53	hsa-miR-221-3p	MPRIP	myosin phosphatase Rho interacting protein
Details	560	53	hsa-miR-221-3p	KIAA1549L	KIAA1549 like
Details	561	53	hsa-miR-221-3p	C12orf77	chromosome 12 open reading frame 77
Details	562	53	hsa-miR-221-3p	PRAMEF13	PRAME family member 13
Details	563	53	hsa-miR-221-3p	NIPA1	NIPA magnesium transporter 1
Details	564	53	hsa-miR-221-3p	RANBP2	RAN binding protein 2
Details	565	53	hsa-miR-221-3p	PRAMEF14	PRAME family member 14
Details	566	53	hsa-miR-221-3p	KCNH1	potassium voltage-gated channel subfamily H member 1
Details	567	53	hsa-miR-221-3p	SLC16A6	solute carrier family 16 member 6
Details	568	53	hsa-miR-221-3p	PCDH11X	protocadherin 11 X-linked
Details	569	53	hsa-miR-221-3p	CHORDC1	cysteine and histidine rich domain containing 1
Details	570	53	hsa-miR-221-3p	PCDH11Y	protocadherin 11 Y-linked
Details	571	52	hsa-miR-221-3p	BCL11B	BCL11B, BAF complex component
Details	572	52	hsa-miR-221-3p	C4orf33	chromosome 4 open reading frame 33
Details	573	52	hsa-miR-221-3p	PRSS37	serine protease 37
Details	574	52	hsa-miR-221-3p	KCNA1	potassium voltage-gated channel subfamily A member 1
Details	575	52	hsa-miR-221-3p	LRP10	LDL receptor related protein 10
Details	576	52	hsa-miR-221-3p	DYRK1A	dual specificity tyrosine phosphorylation regulated kinase 1A
Details	577	52	hsa-miR-221-3p	SLAIN2	SLAIN motif family member 2
Details	578	52	hsa-miR-221-3p	DDX58	DExD/H-box helicase 58
Details	579	52	hsa-miR-221-3p	TAF9B	TATA-box binding protein associated factor 9b
Details	580	52	hsa-miR-221-3p	CYP1B1	cytochrome P450 family 1 subfamily B member 1
Details	581	52	hsa-miR-221-3p	UBN2	ubinuclein 2

Details	582	52	hsa-miR-221-3p	GPATCH2L	G-patch domain containing 2 like
Details	583	52	hsa-miR-221-3p	SNCA	synuclein alpha
Details	584	52	hsa-miR-221-3p	WDR77	WD repeat domain 77
Details	585	52	hsa-miR-221-3p	IL1RAP	interleukin 1 receptor accessory protein
Details	586	51	hsa-miR-221-3p	CHFR	checkpoint with forkhead and ring finger domains
Details	587	51	hsa-miR-221-3p	C16orf82	chromosome 16 open reading frame 82
Details	588	51	hsa-miR-221-3p	ZBTB20	zinc finger and BTB domain containing 20
Details	589	51	hsa-miR-221-3p	ESYT1	extended synaptotagmin 1
Details	590	51	hsa-miR-221-3p	KRT81	keratin 81
Details	591	51	hsa-miR-221-3p	DICER1	dicer 1, ribonuclease III
Details	592	51	hsa-miR-221-3p	MDM2	MDM2 proto-oncogene
Details	593	51	hsa-miR-221-3p	ZBTB41	zinc finger and BTB domain containing 41
Details	594	51	hsa-miR-221-3p	NCAM1	neural cell adhesion molecule 1
Details	595	51	hsa-miR-221-3p	MAP4K4	mitogen-activated protein kinase kinase kinase kinase 4
Details	596	51	hsa-miR-221-3p	MYOD1	myogenic differentiation 1
Details	597	51	hsa-miR-221-3p	GNB3	G protein subunit beta 3
Details	598	51	hsa-miR-221-3p	MMD2	monocyte to macrophage differentiation associated 2
Details	599	51	hsa-miR-221-3p	SLC25A21	solute carrier family 25 member 21
Details	600	50	hsa-miR-221-3p	ITPRID1	ITPR interacting domain containing 1
Details	601	50	hsa-miR-221-3p	CAVIN4	caveolae associated protein 4
Details	602	50	hsa-miR-221-3p	AGO4	argonaute RISC catalytic component 4
Details	603	50	hsa-miR-221-3p	AJAP1	adherens junctions associated protein 1
Details	604	50	hsa-miR-221-3p	ITIH5	inter-alpha-trypsin inhibitor heavy chain family member 5
Details	605	50	hsa-miR-221-3p	CNKSR3	CNKSR family member 3
Details	606	50	hsa-miR-221-3p	CT45A5	cancer/testis antigen family 45 member A5
Details	607	50	hsa-miR-221-3p	PKDCC	protein kinase domain containing, cytoplasmic
Details	608	50	hsa-miR-221-3p	STAT2	signal transducer and activator of transcription 2
Details	609	50	hsa-miR-221-3p	A4GNT	alpha-1,4-N-acetylglucosaminyltransferase
Details	610	50	hsa-miR-221-3p	CT45A10	cancer/testis antigen family 45 member A10
Details	611	50	hsa-miR-221-3p	GCNT1	glucosaminyl (N-acetyl) transferase 1
Details	612	50	hsa-miR-221-3p	ERI2	ERI1 exoribonuclease family member 2
Details	613	50	hsa-miR-221-3p	APAF1	apoptotic peptidase activating factor 1
Details	614	50	hsa-miR-221-3p	CLDN12	claudin 12
Details	615	50	hsa-miR-221-3p	SAR1A	secretion associated Ras related GTPase 1A
